# Computational modeling of bone allograft reconstruction following femoral shaft tumor resection: Investigating the impact of supplementary plate fixation

**DOI:** 10.1371/journal.pone.0316719

**Published:** 2025-02-06

**Authors:** Petr Boháč, Vasileios Apostolopoulos, Petr Marcián, Tomáš Tomáš, Michal Mahdal, Tomáš Návrat

**Affiliations:** 1 Faculty of Mechanical Engineering, Institute of Solid Mechanics, Mechatronics and Biomechanics, University of Technology, Brno, Czech Republic; 2 First Department of Orthopaedic Surgery, St. Anne’s University Hospital and Faculty of Medicine, Masaryk University, Brno, Czech Republic; The Affiliated Changzhou No 2 People’s Hospital of Nanjing Medical University, CHINA

## Abstract

**Background and objective:**

The use of bone allograft reconstructions after tumor resection can introduce significant complications. Stable fixation is required to decrease the incidence of mechanical complications of segmental bone allografts. The purpose of the present study is to compare plating fixation methods of diaphyseal allografts after intercalary resection of the femur.

**Methods:**

We created four defined fixation models using plates and/or intramedullary polymethylmethacrylate (PMMA) to simulate typical bone tumor resection with intercalary allograft reconstruction. One angularly stable plate (DFP) with 13 locking screws and fresh frozen allografts (labeled “I”) were used for bone reconstruction. Three modified reconstructions were created: “II” included a supplementary plate (SP) with four locking screws, “III” was augmented with intramedullary PMMA in the allograft, and “IV” combined intramedullary PMMA and both plates. We applied a load model that simulates partial weight bearing on the lower limb to simulate the load during postoperative rehabilitation.

**Results:**

The highest stress in the DFP occurred at the allograft-bone transition, with variant IV reaching 297 MPa. PMMA augmentation reduced median interfragmentary motion (IFM) and sliding distances, with variant III achieving the lowest distal sliding distance (0.9 μm) in the distal area. Supplementary plate fixation reduced maximal and median proximal IFM distances (86.9 μm in variant II vs. 116.0 μm in variant I) but increased sliding distances (23.7 μm in variant II vs. 0.6 μm in variant I).

**Conclusions:**

PMMA augmentation reduces IFM and sliding distances, enhancing rigidity, particularly in the distal area. Supplementary plate fixation decreases IFM distances in the proximal area but increases sliding distances in the same region. Variants III and IV demonstrate lower IFM and sliding distances in the distal area overall. Variant III shows very low sliding distances in both distal and proximal areas. Variant IV combines improved firmness with slightly higher stress levels.

## 1. Introduction

Limb salvage surgery is the method of choice in the treatment of malignant bone tumors. Surgical removal is still the primary and most effective therapy for bone sarcomas [1, 2]. A precise *en bloc* surgical resection is crucial to achieve adequate margins without penetrating the tumor at the cost of large bone loss [3, 4]. The treatment of large bone defects remains one of the main challenges in orthopedics. Progress in oncological therapies has led to increased patient survival and, consequently, the need for long-term bone defect reconstruction [5–7].

The optimal reconstruction method after intercalary resection of diaphyseal tumors remains unsettled. The introduction of intercalary endoprostheses capable of reconstructing large diaphyseal defects allows early mobilization and offers good function and mechanical support [5, 8–10]. However, there are some instances where an intercalary endoprosthesis may not be the preferred option [8, 11]. Implantation of an intercalary endoprosthesis is relatively simple, but its long-term survival is still of great concern, and this factor limits its acceptance. Cortical allografts restore bone stock, promote improved soft-tissue attachment, and can survive for a long period of time compared with metal implants [5, 8]. Despite these benefits, the utilization of large segmental bone allografts can introduce significant complications. Among the most frequently encountered complications after the operation are hardware, allograft, and host bone failure, and consequent non-union [9, 12].

Stable fixation is required to decrease the incidence of mechanical complications of segmental bone allografts. The fixation technique can vary depending on the length and the anatomical location of the diaphyseal defect [5]. These are still concerns about the optimal fixation of segmental bone allografts to achieve uncomplicated ingrowth and bone union [7, 9, 13]. Common methods of allograft fixation include extramedullary fixation with a single plate or multiple plates [6, 14]. An additional fixation method for plating is the use of intramedullary PMMA, also known as bone cement. The use of cemented allografts is thought to decrease complications associated with implant loosening or allograft failure, but it may slightly delay allograft healing [14–16]. During polymerization, PMMA generates heat, which can lead to thermal necrosis in the surrounding allograft bone and residual stresses in PMMA itself. Balancing PMMA’s mechanical benefits with its biological drawbacks is crucial for achieving better outcomes in PMMA augmentation.

The purpose of the present study was to compare plate fixation methods of diaphyseal allografts after intercalary resection of the femur. A finite element method (FEM) simulation approach was chosen to investigate the optimal fixation method. There were created femur models after intercalary resection with a segmental bone allograft (allogenous fresh frozen cortical bone) of four defined fixation methods using plates and/or intramedullary PMMA. We hypothesize that the combination of multiple plates with intramedullary PMMA provides an enhanced immediate clinical environment conducive to healing for the reconstruction of diaphyseal allografts following intercalary resection. Our study could provide additional evidence to prevent complications associated with implant loosening or allograft failure.

In the field of orthopedics, computational modeling represents a useful tool for numerous applications [17–19]. It can help to simulate and analyze complex biomechanical states in the human body, which could be very challenging or even almost impossible to replicate experimentally [20]. One of the most significant impacts can be in preoperative planning, where typical patient computational models based on computed tomography (CT) data allow the simulation of an orthopedic surgery procedure [21, 22]. Analysis of the data allows for more informed decisions about implant placement, alignments, or selection [23].

## 2. Materials and methods

### 2.1 Surgical technique

The surgical treatment technique for primary bone sarcoma involves an *en bloc* resection of the tumor followed by reconstruction using an intercalary bone allograft. A lateral approach to the femur is preferred, ensuring optimal access for the procedure. The surgical process begins with the precise resection of the lesion, ensuring adequate margins are achieved. All allografts are maintained under sterile conditions and stored frozen at -80°C in a specialized bone bank. Upon thawing in a warm solution, a fresh deep-frozen allograft segment, sized to fit the bone defect, is cut to the proper length. All allograft-host junctions are performed with a transverse osteotomy. During this stage, the option to augment the intercalary allograft cavity with PMMA is considered to enhance stability and support. Subsequently, osteotomies were secured using a long plate with locking screws to achieve a robust fixation and safeguard the integrity of the entire graft. Depending on the specific anatomical site and requirements, additional stabilization with a short supplementary plate or multiple plates could be implemented to further reinforce the osteotomy site and support bone healing.

### 2.2 Geometry models

CT scans of both femurs and the pelvis were obtained with a CT scanner (GoldSeal Optima CT660, GE HealthCare, CI, USA) with a voxel size of 0.9336 **×** 0.9336 **×** 0.625 mm^3^. In an application programmed in the MATLAB 2012 environment (Math Works, Natick, MA, USA) [24], these CT scans were manually segmented, and the result was Standard Tessellation Language (STL) files containing the surface geometry. Subsequently, all STL files were modified and converted to solid geometry in SpaceClaim ANSYS® Academic Research Mechanical, Release 22.2 (Swanson Analysis, Inc., Houston, PA, USA). The volume geometries of the technical components were also modified in this program by replacing the threads in the fixation plates and screw heads with conical surfaces [17, 25].

The geometry models of the femur, pelvis, allograft, distal femoral angularly stable plate (DFP), screws, and a straight angularly stable plate (SP) or bone cement were included in the computational models. The DFP measures 299 mm in length, with 12 holes in the proximal region and a thickness of 6 mm (cat. no. 397129779620), while the SP is 105 mm long, 16.2 mm wide, with 6 holes and a thickness of 5 mm (cat. no. 39712977350). The geometry models of the technical components were obtained from the manufacturer (MEDIN, a.s., Nové Město na Moravě, Czech Republic). The femur geometry and material properties distinguish cortical and cancellous bone tissues.

**Fig 1:**
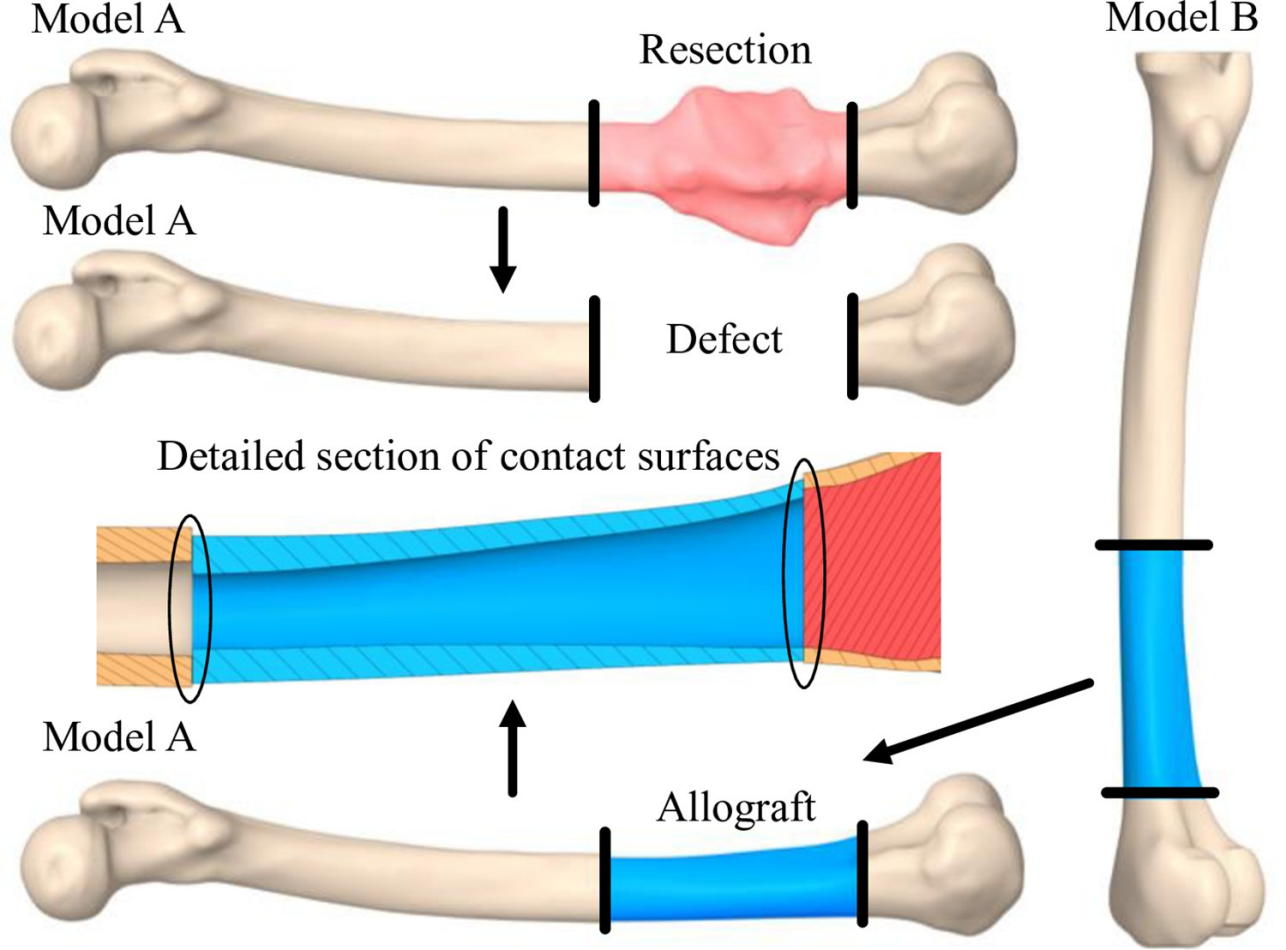
Resection of the bone tumor of femoral bone model A followed by reconstruction of the resultant defect using the allograft from femoral bone model B process.

The femur bone tissue models were created from CT scans of two patients, labeled “A” and “B.” Model “A” was created from CT scans of a representative patient diagnosed with osteosarcoma of the femur; in this case, the osteolytic lesion did not involve the knee joint. Model “B” was created from CT scans of a representative patient that corresponds proportionally to the first patient. The resulting geometry models were created by using the described procedure to simulate a typical bone tumor resection with intercalary allograft reconstruction. A 120 mm long part in the area of the distal femur with a tumorous lesion was removed from the femur “A” model to simulate an intercalary *en bloc* bone resection. The defect in the femur “A” model was replaced with a corresponding part of the same length from the femur “B” model to simulate an allograft. This surgical procedure is demonstrated in Fig 1. Then, femur model “A” was positioned to achieve a small contact area between the allograft from femur “B” from both sides. These two contact surfaces are located at the outer edges of the cortical bone tissue models at the distal femoral plate region.

Furthermore, a model of the pelvis was created by using CT scans of patient “A” and positioned physiologically. The contact surfaces of the hip were adjusted to a perfect sphere, and two cartilage models, each with a thickness of 2 mm, were placed on these surfaces [26]. The resulting assembly with cartilages is shown in Fig 3.

**Fig 2:**
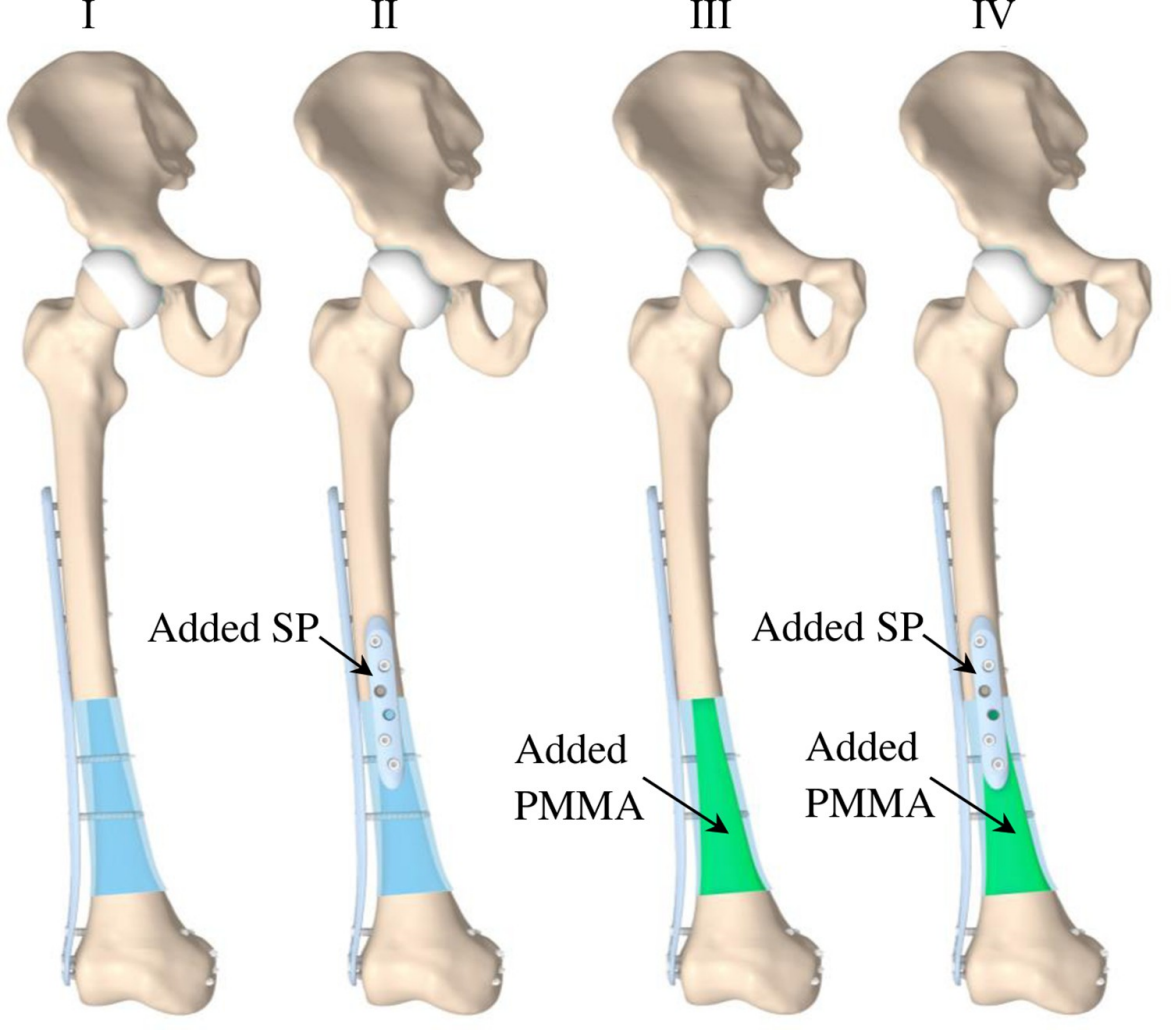
The final assemblies of the geometry models. Reconstruction I is stabilized with a single DFP. Reconstruction II incorporates additional supplementary fixation with SP. Reconstructions III and IV are augmented with intramedullary PMMA in the allograft. Reconstruction III is stabilized with a single DFP, and reconstruction IV has additional supplementary fixation with SP.

One DFP and 13 locking bone screws (HA 5), each with a diameter of 5 mm but a variable length of 30–95 mm (standardized lengths graduated in 2 or 5 mm increments), were added to this assembly according to the principles of Arbeitsgemeinschaft für Osteosynthesefragen (AO) [27] and of fresh frozen allografts used for bone reconstruction, as described in numerous studies [8, 9, 28–30]. The final assembly is shown in Fig 2, labeled “I.” In addition, three modified reconstructions were created, the first modified reconstruction is labeled “II”; it has an added SP and four locking self-tapping screws. Of them, two screws were placed into the allograft and two into the femur bone. The second modified reconstruction, labeled “III,” is augmented with intramedullary PMMA in the allograft. The third modified reconstruction (labeled “IV”) is augmented with intramedullary PMMA in the allograft and both plates as presented in the reconstruction “II.” In total, four different assemblies were created.

### 2.3 Meshing procedure

All solid models underwent discretization in ANSYS®, using quadratic hexahedral and quadratic tetrahedral elements, specifically the SOLID186 and SOLID187 element types. The contact surfaces were meshed with the contact elements CONTA174 and TARGE170. For reconstructions I and III, the mesh contained approximately 19 million elements, while for reconstructions II and IV, the mesh contained approximately 27 million elements. The global element sizes were set at 1 mm for cortical bone tissues and at 2.5 mm for cancellous bone tissues and bone cement. Both fixation plates were set to 1 mm, and all of the screws were meshed in the range of 0.05 mm on the threads to 0.4 mm globally. The mesh was also adjusted on the contact surfaces around the allograft to 0.25 mm. Cartilages were meshed with an element size of 1 mm. The above-mentioned element sizes were determined through preliminary tests and sensitivity calculations.

For the meshing procedure and computation, the following hardware was utilized: an AMD Ryzen CPU at 3.8 GHz with 24 cores, 512 GB of DDR4 RAM, an NVIDIA T1000 GPU, an 8 TB SSD, and Windows Server as the operating system.

### 2.4 Material models

All materials were assumed to be linearly elastic, isotropic, and homogeneous based on Young’s modulus and Poisson’s ratio. The material properties were selected carefully based on the related previous studies (Table 1). Based on prior research [29], the allograft was considered to be as mechanically competent as healthy cortical bone tissue.

**Table 1:** The material properties for each part of the assembly.

Part	Material	E [MPa]	μ [–]	Reference
Fixation plates, screws	Stainless steel (316L)	195 000	0.30	[31]
Femur, Allograft	Cortical bone	17 600	0.30	[32, 33]
Femur	Cancellous bone	350	0.25	[33]
Pelvis	Cortical bone	17 000	0.30	[34]
Bone cement	Palacos®	2 891	0.40	[35]
Cartilages	Cartilage	12	0.45	[33]

### 2.5 Loads and boundary conditions

**Fig 3:**
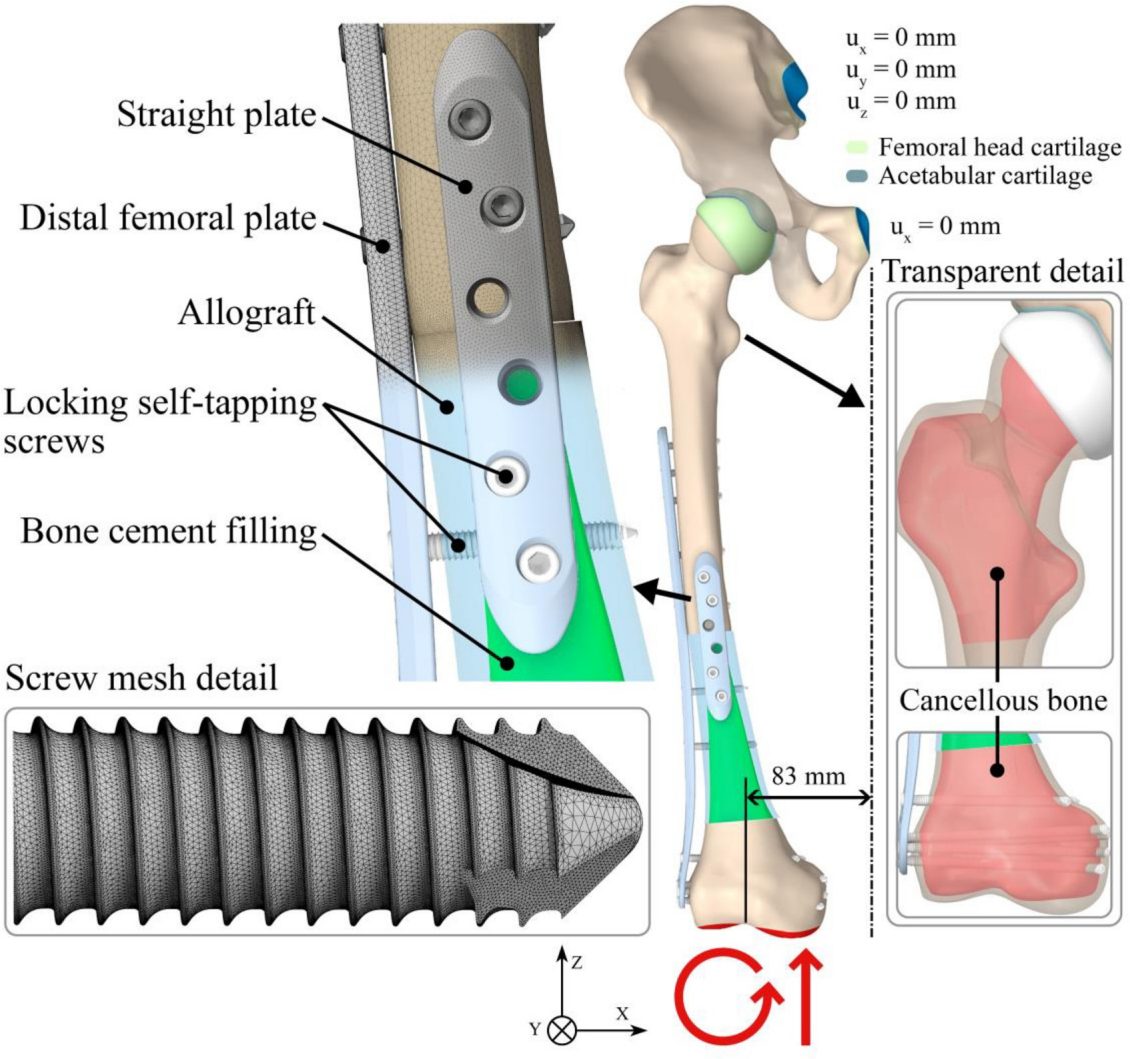
The geometry model of reconstruction variant IV with the boundary conditions and parts description. Additional transparent detail with cancellous bone geometry model, and hip cartilage.

The load model is based on the static balance of a person standing straight on both legs. The patient’s body weight (BW) is assumed to be 80 kg. The load during postoperative rehabilitation is simulated by applying a load model that models partial weight bearing (50% of total BW) on the lower limb. The values are presented as multiples of BW by a factor of g (g = 9.81 ms^-2^). Therefore, the load is applied to the outer surfaces of both condyles of the femur as an acting force in the direction of the “z” axis, and the moment around the “y” axis. A boundary condition preventing displacements in all directions in the region sacroiliac joint is applied to the pelvis, and a boundary condition preventing displacements in the direction of the “z” axis is applied in the region of symphysis pubis [36]. Fig 3 shows the prescribed boundary conditions. Table 2 shows the applied forces and moments. The load model is the same for all four variants.

**Table 2:** The applied forces and moments.

Axis	Force [N]	Moment [N·m]
Z	- 0.5 × BW	0
Y	0	-0.5 × BW × 0.083

**Fig 4:**
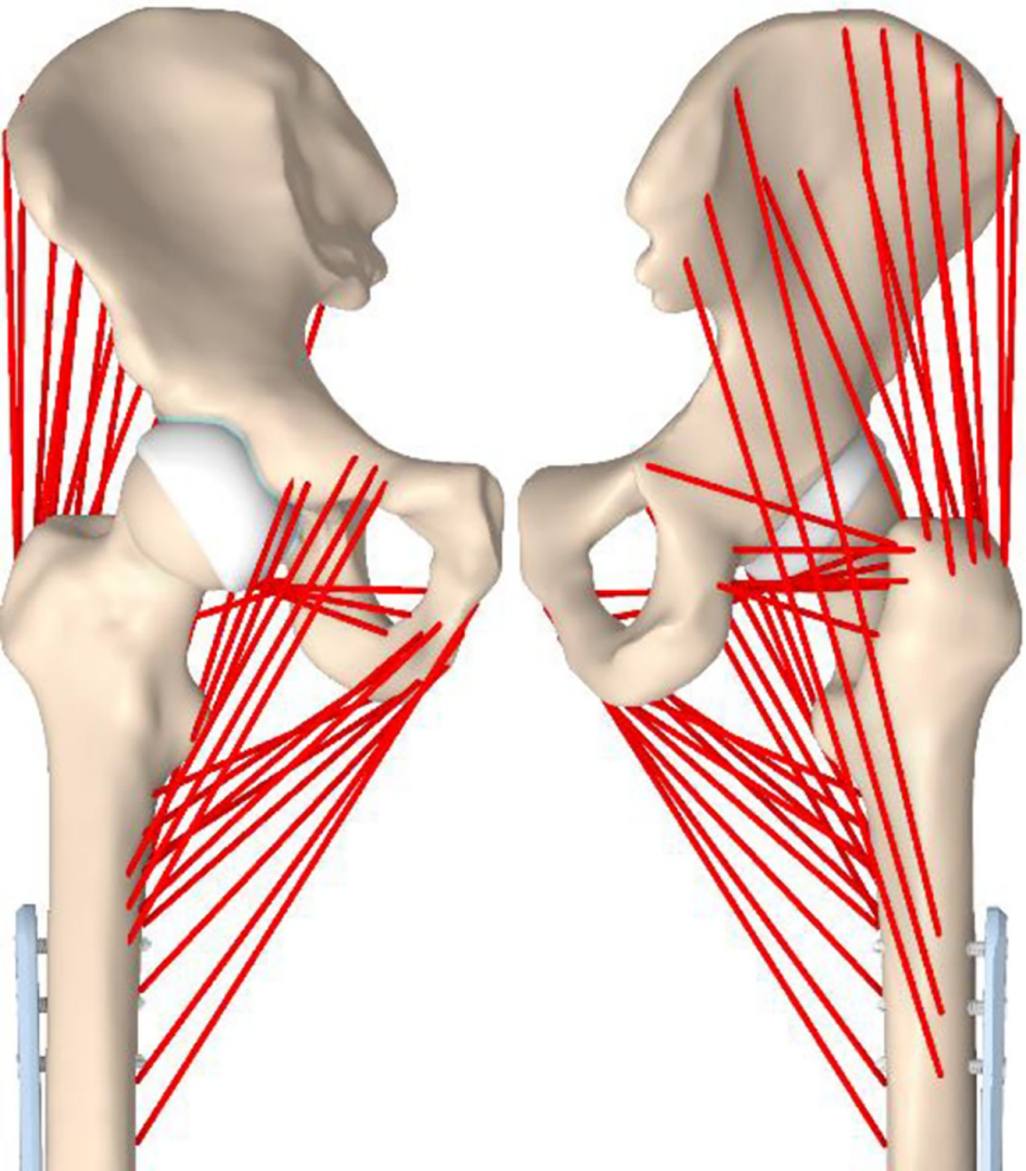
An illustration of the muscle model from frontal and dorsal view.

The muscle model was created in ANSYS by using 14 springs attached to their anatomical positions. It is assumed that all muscles are in isometric contraction, that is, the tension increases without a significant change in length [37]. The springs are set to work in pull mode only. The isometric stiffness (k_iso_) of the springs, which represents muscles, is based on the FEM analysis [38]. For this condition, ANSYS uses the LINK180 element in the “tension only” mode in the calculation. The muscle representations and their isometric stiffness values are listed in Table 3. Fig 4 shows the muscle representations that were created and used in this analysis.

**Table 3:** The applied forces and moments for the muscles.

Muscle	k_iso_ [N/mm]	Muscle	k_iso_ [N/mm]
Gluteus minimus	660	Adductor longus	134
Gluteus medius	779	Iliacus	167
Gluteus maximus	344	Psoas	100
Piriformis	90	Adductor brevis	499
Gemellus superior	49	Quadratus femoris	372
Gemellus inferior	49	Pectineus	306

Note. Values from article A.T.M. Phillips, P. Pankaj, C.R. Howie, A.S. Usmani, A.H.R.W. Simpson, Finite element modelling of the pelvis: Inclusion of muscular and ligamentous boundary conditions, Medical Engineering & Physics 29 (2007) 739–748. https://doi.org/10.1016/j.medengphy.2006.08.010.

In the context of hardware such as screws and fixation plates, all interactions with cortical bone, cancellous bone, and bone cement are set as frictional contact. The screw thread geometry model was used as provided by the manufacturer, accurately reflecting the real shape. The interfaces between articular cartilage are also set as frictional contact. However, the cortical bone and cartilage, specifically in the femoral head and pelvis, are rigidly connected. The contact between the cancellous bone and cortical bone inside the femur is set as rigidly connected. The contact between the allograft and bone cement is set as rigidly connected. All of the contacts on the newly formed straight-cutting faces of the allograft and original femur are set as frictional. The friction coefficients are presented in Table 4.

**Table 4:** The applied friction coefficients to the surface pairs.

Contact surfaces	Friction coefficient [–]	Reference
Steel for implant–cortical bone	0.37	[39]
Steel for implant–cancellous bone	0.25	[40]
Steel for implant–bone cement	0.54	[41]
Cortical bone–cortical bone (allograft)	0.58	[42]
Cortical bone (allograft)–cancellous bone	0.58	[42]
Cortical bone–bone cement	0.60	[43]
Cancellous bone–bone cement	0.60	[43]
Cartilage–cartilage	0.005	[44]

## 3. Results

### 3.1 Equivalent stress

#### 3.1.1 Fixation plates

**Fig 5:**
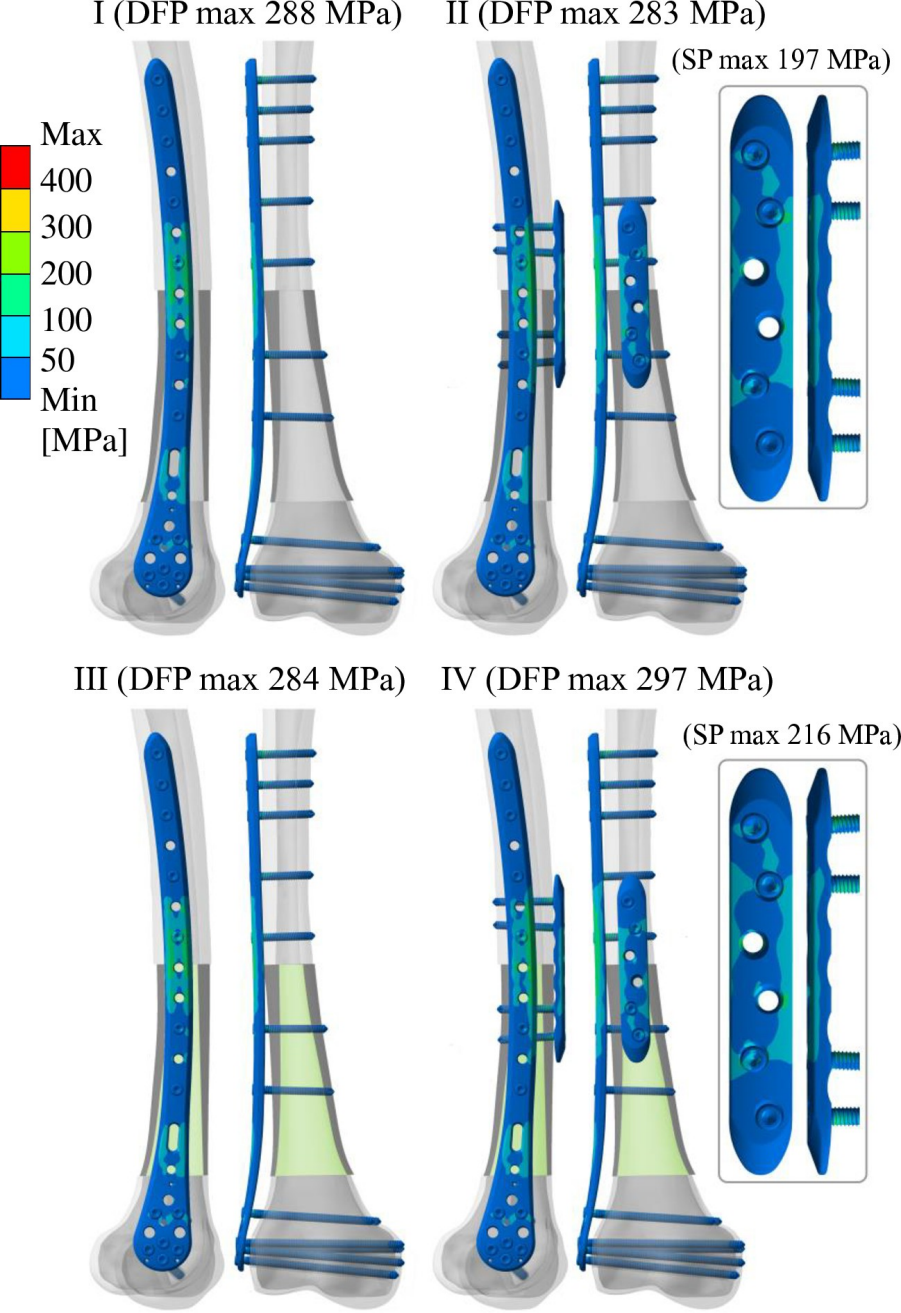
The equivalent stress distribution for each analyzed variant in the frontal and sagittal views with additional details of the straight plate.

Fig 5 shows the equivalent (von Mises) stress distribution in the fixation plates and screws for each analyzed variant. The allograft is highlighted by the darker gray color, and augmentation with PMMA is highlighted by the light green color. For the four analyzed variants, the highest equivalent stress in the DFP is in the distal transition between the allograft and the original bone. The maximum equivalent stress in the DFP is 297 MPa for variant IV. The maximum equivalent stress in the SP is 216 MPa for variant IV; variant II has a maximum equivalent stress in the SP of 197 MPa.

#### 3.1.2 PMMA

The equivalent stress distribution in PMMA for variant II shows the highest value of 7.9 MPa, it is located in a small number of elements on the edge of the proximal end closer to the distal plate, where is initiated frictional contact with cortical bone tissue. The rest of the higher values around 4 MPa are located around the screw threads. In variant IV maximum equivalent stress of 23 MPa is in a small number of elements around the sharp edge of one of the screws thread interface and allograft interface. The equivalent stress values are higher for the screws associated with the SP than for the DFP. Some additional higher values of equivalent stress are located also in the proximal end around the edge of the geometry. The values range from 10 to 14 MPa. All the values of the equivalent stress are below the 40 MPa yield strength of the PMMA [45].

#### 3.1.3 Locking screws

The analysis of the equivalent stress distribution on the screws has shown a phenomenon of stress singularities around the “bonded” contact between the fixation plates and screw heads, where the locking mechanism is replaced by the conical surfaces. This phenomenon is appearing on every screw and every variant where the chamfer is located. For the DFP screws, the highest equivalent stress value is 569 MPa, and for SP screws it is 815 MPa. From the mesh sensitivity testing, we have analyzed that this phenomenon is amplified when finer mesh is used. If we do not consider this phenomenon, the highest value of the equivalent stress is appearing around the neck of the screws, and the first few threads range from 195–390 MPa for DFP screws and 200–505 MPa for SP screws. Where the maximum yield strength of the material is 890 MPa [46].

### 3.2 Interfragmentary motion distance and sliding distance of the allografts

To analyze the mutual motion between the original bone and the allograft, we adopted the current comprehensive approach developed by Sun et al. [20] for similar orthopedic cases. We used the IFM-Cal program, created in Python 3.10.11, to evaluate the interfragmentary motion (IFM) distance and sliding distance. Proper data preparation in the Ansys environment is required to use this program effectively. We believe that the results from IFM-Cal will offer more comprehensive and accurate evaluations for our study.

**Fig 6:**
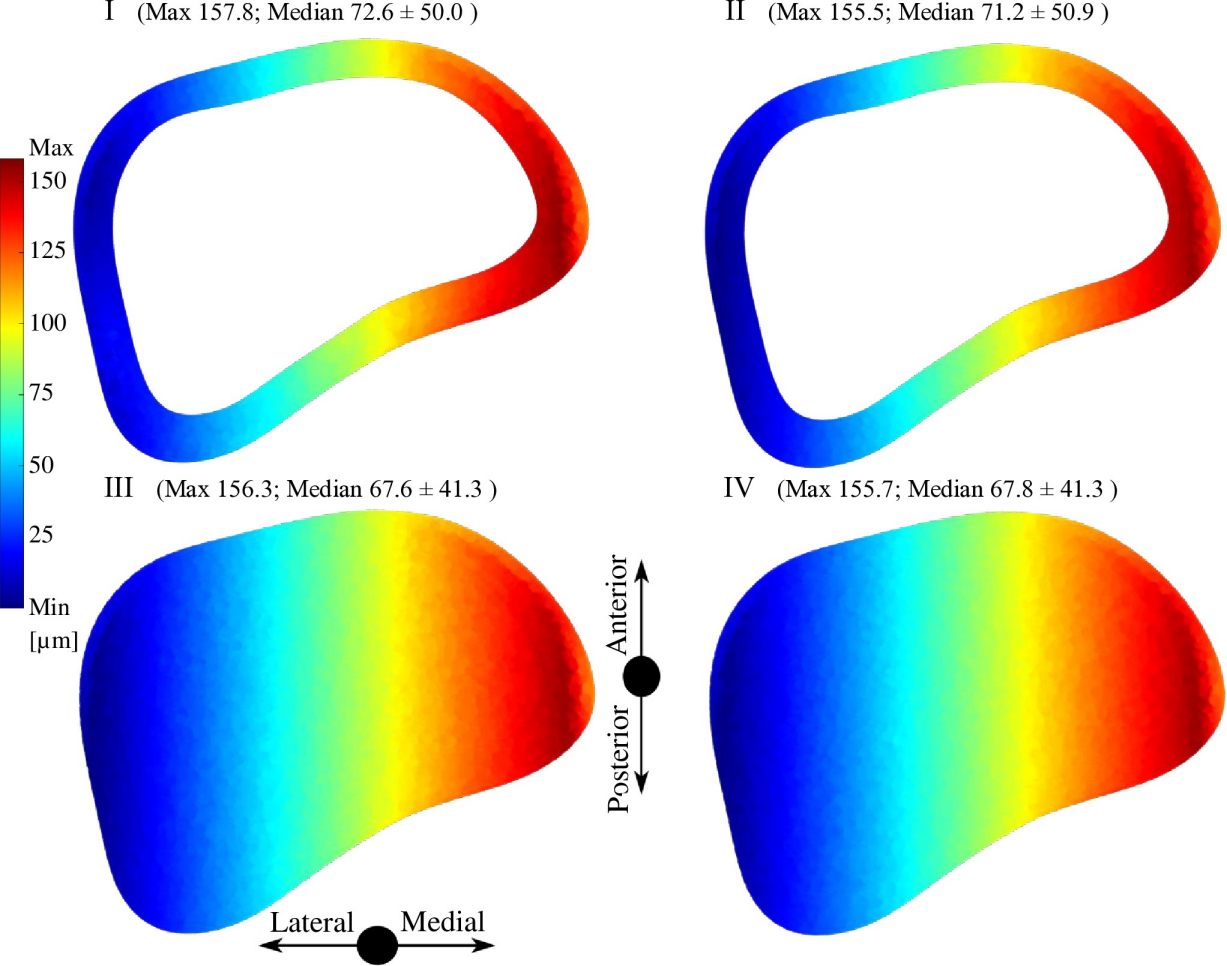
IFM distance distribution for the four analyzed variants in the distal area.

**Fig 7:**
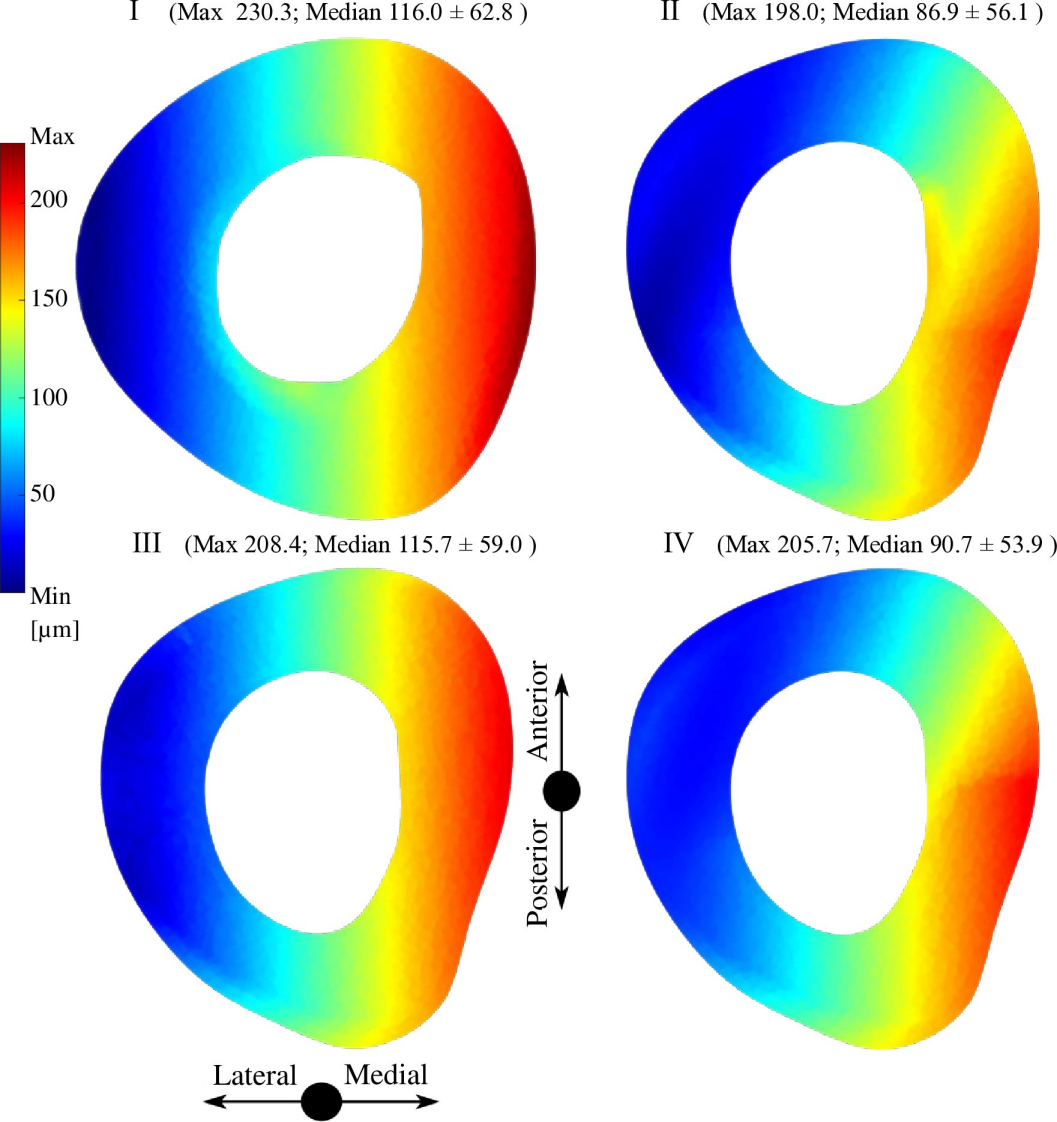
IFM distance distribution for the four analyzed variants in the proximal area.

**Fig 8:**
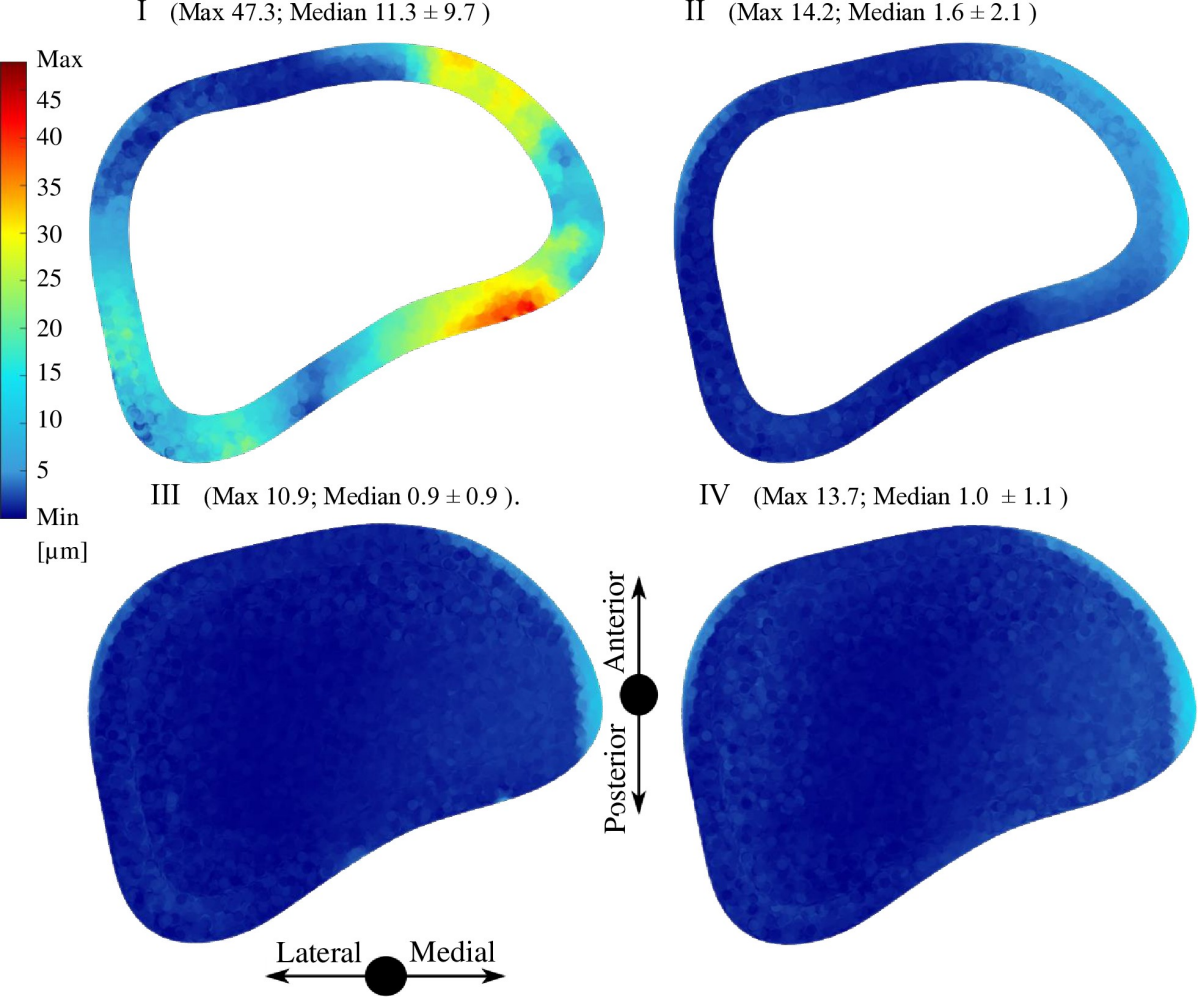
Sliding distance distribution for the four analyzed variants in the distal area.

**Fig 9:**
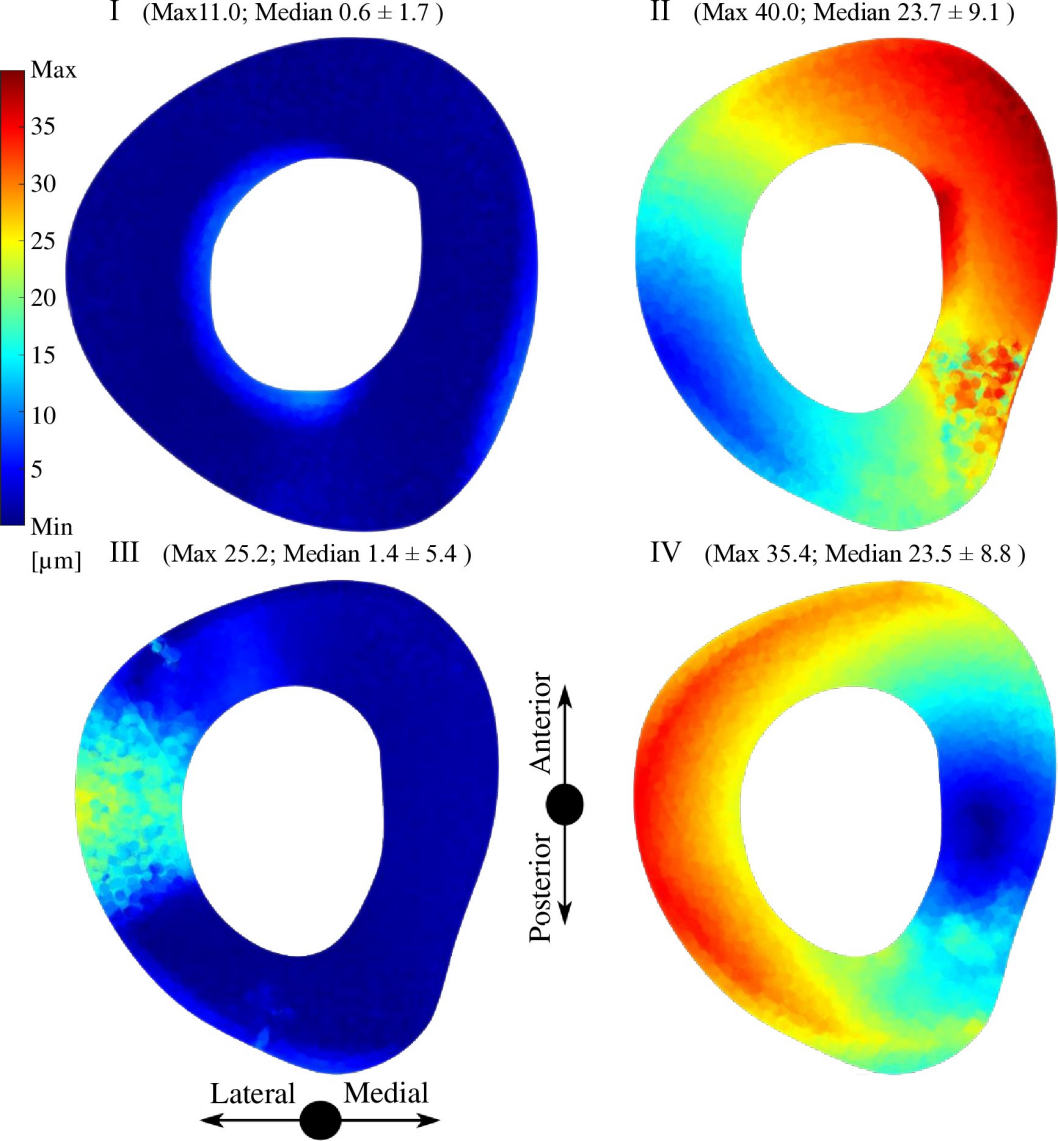
Sliding distance distribution for the four analyzed variants in the proximal area.

The IFM distance represents the axial and tangential motion of the analyzed parts referred to the local coordinate system positioned in the middle of the surface of the allograft on the distal and proximal part (according to guidelines of authors of the IFM-Cal program). The sliding distance represents only the tangential part of the motion. All details about the IFM and sliding distance are presented in the study of Sun et al. [20]. Figs 6 and 7 show the interfragmentary motion (IFM) distance between the allograft and the original bone tissue in the transversal plane. Figs 8 and 9 show of sliding distance between the allograft cortical bone tissue and the original cortical bone tissue.

#### 3.2.1 Distal area

The maximum IFM distance, approximately 158 μm, is observed in variant I. While there are minimal differences in the maximum IFM distance among the variants, there are notable differences in median IFM distances between variants with PMMA (III, IV) and without PMMA (I, II) augmentation. Specifically, the median IFM distance is 67.6 μm for variant III and 67.8 μm for variant IV, whereas 72.6 μm for variant I and 71.2 μm for variant II. For variants III and IV, there is an additional contact area between the intramedullary PMMA and the cancellous bone tissue.

Among the four analyzed variants, there are notable differences in sliding distance (tangential motion) values and distribution. The highest values of sliding distance of 47.3 μm appear in variant I, together with the highest median value of 11.3 μm. A minimum sliding distance of 10.9 μm appears in variant III, together with the minimum median sliding distance of 0.9 μm. Variants II, III, and IV exhibit comparable maximum and median values of sliding distance, with consistent localization patterns. In contrast, variant I shows notable differences in both the magnitude of values and their localization.

#### 3.2.2 Proximal area

There are no influential differences between variants I and III regarding the median IFM distance values and distribution. The highest value of IFM distance is 230.3 μm and appears in variant I. There is a visible difference in the distribution of IFM distance between variants I, II (without PMMA) and II, IV (with PMMA). When examining the median values, variants II and IV which include supplementary SP, exhibit smaller values of IFM distance compared to variants I and III without SP. Specifically, variants I and III with median IFM distances of 116.0 μm and 208.4 μm respectively, contrasted with variant II with a median IFM distance of 86.9 μm and variant IV with a median IFM distance of 90.7 μm.

Among all analyzed variants there are visible differences in the values and distribution of the sliding distance. Variants I and III without supplementary SP have considerably smaller maximum and median sliding distance values compared to variants II and IV with additional SP. Specifically, variant I and variant III exhibit maximum sliding distances of 11.0 μm and 25.2 μm, respectively, compared to variants II and IV, which show maximum sliding distances of 40.0 μm and 35.4 μm, respectively.

## 4. Discussion

Bone allograft replacement is a standard treatment method for diaphyseal defects following bone tumor *en bloc* resection [30]. It is a biological reconstruction with the aim of restoring the continuity of the bone with new bone tissue from the same patient, which is an advantage over endoprosthetic reconstruction in young patients [8, 28]. However, the technique is not suitable when used for a patient with limited survival. Many bone allograft reconstruction techniques have been described, and researchers have investigated the clinical outcomes [8, 12]. Cortical allografts still present high rates of mechanical complications, and the optimal method of reconstruction remains unsettled [9, 10]. There are no evidence-based guidelines for orthopedic surgeons to consider for cortical allograft reconstruction, especially in terms of biomechanical analysis. In the present study, we investigated the optimal type of reconstruction after intercalary resection with a segmental bone allograft by comparing four defined fixation methods using plates and/or intramedullary bone cement.

Various studies have consistently recorded superior outcomes with plate osteosynthesis over fixation with intramedullary nails [8, 9, 16]. For example, plate osteosynthesis is associated with a lower risk of malunion. However, plate fixation comes with its own set of challenges, such as a higher likelihood of hardware fracture and an increased risk of allograft fracture at the site of screw insertion in the diaphyseal parts [8, 16]. Frisoni et al. [9] evaluated bone healing factors of intercalary allografts and specifically cautioned against the use of intramedullary nails due to their tendency to lead to malunion; they recommended the use of plates as a more reliable alternative. Additionally, Muscolo et al. [47] suggested a combination of two plates applied orthogonally or in parallel for cases of long allograft reconstructions. In our femur model, the orthogonal application of plates appears to be the optimal option from the perspective of the femur lateral surgical approach. Other authors have recommended intramedullary allograft PMMA augmentation to reduce fracture and infection rates [15, 48–50]. There is ongoing controversy regarding the use of PMMA for augmenting bone allografts. However, the literature suggests that adding intramedullary cement to large-segment bone allografts can enhance their survival by reducing the risk of fracture, particularly in intercalary reconstructions [48, 51, 52]. Although filling the bone marrow cavity with PMMA can disrupt endosteal bone formation, most new bone growth originates from the periosteal surfaces rather than the endosteal surfaces of allograft cortical bone. This implies that intramedullary PMMA augmentation may not significantly impact the healing process [53]. Additionally, PMMA should be loaded with antibiotics, such as gentamicin, to prevent infections, which are the most common complications associated with bone allografts [15]. The comparable stress levels observed across fixation variants in our study reinforce the reliability of plate-based methods, as supported by prior research highlighting superior outcomes of plate osteosynthesis over intramedullary nails due to a lower risk of malunion. While plate fixation carries risks such as hardware and allograft fractures at screw sites, our findings suggest that PMMA augmentation effectively mitigates these challenges by enhancing rigidity and reducing distal movement. This supports Muscolo et al.’s recommendation for optimized plate configurations and aligns with the broader literature advocating combined plate fixation as the preferred approach for femoral shaft allograft reconstruction after tumor resections.

In the present study, the four analyzed variants show a comparable equivalent stress level in the DFP. The highest equivalent stress in the DFP occurs in the distal transition between the allograft and the original bone. For variant IV, the highest equivalent stress in the DFP is 297 MPa, which approaches the yield strength of 890 MPa [46] with a safety factor of 3. DFP failure is not expected in any of the examined reconstructions. Augmentation with PMMA has a minor effect on equivalent stress in variant III without additional SP. On the other hand, augmentation with PMMA slightly increased maximum equivalent stress in both SP and DFP for variant IV. The maximum equivalent stress of 505 MPa in variant IV occurs on the first few threads of an SP screw, still below the material’s yield strength of 890 MPa [46], with a safety factor of 1.76. While the yield strength of the plates was not exceeded under a single load, it is important to consider that repeated loading occurs during daily activities. During the postoperative rehabilitation period (up to 6 months), patients are restricted to partial weight bearing (50% of total body weight) using crutches, and as bone healing progresses, the stability of the reconstruction improves. Our simulations represent the maximum load that could occur immediately after surgery, prior to bone healing, and over time, we expect the stresses on the material to decrease as healing advances.

The lack of stability allowing junction movements is one of the main factors that contributes to the failure of the junction between the original bone and the allograft. Rigid fixation enhances bone union and helps to achieve bone healing [8, 16, 54]. In our study, we have found that the IFM distance for all the variants is within the acceptable ranges to promote bone healing. The influence of PMMA augmentation is most evident in the distal area, where it results in smaller median IFM distance values. The addition of SP fixation further reduces the median IFM distance values but increases both the median and maximum sliding distance values in the proximal area. In the distal area, the IFM distance and sliding distance are comparable across the variants with PMMA. Among all variants, variant III demonstrates the lowest IFM and sliding distance values in the distal area, with a median sliding distance value of only 1.4 μm in the proximal area. The sliding distance values observed in our study align with the interfragmentary motion ranges documented in prior research. Kenwright and Goodship recommended an optimal interfragmentary motion of 0.2–1.0 mm for promoting uncomplicated bone healing [55]. Similarly, Wolf et al. proposed an interfragmentary motion within the range of 0.4 mm [56]. Unfortunately, previous studies have not specifically analyzed the motion ranges at the allograft-bone interface.

The present study has several limitations. First, the analysis is limited to a single anatomical location (distal femoral diaphysis) and a specific defect length (120 mm). Second, the load model simulates axial weight bearing on the limb and lacks torsional load. Although we set the hardware position according to the principles of AO and of fresh frozen allografts for bone reconstruction, there is a wide variety of possible screw positions and quantities that could be tested. Additionally, only materials defined by homogeneous and linear behavior were employed in the computational models. Nonetheless, this is a highly specialized surgical procedure (orthopedic oncology), and there is an absence of biomechanical evidence regarding the options of bone allograft fixation. Furthermore, we simulated various fixation methods using patient-specific computational models derived from CT scans, incorporating a detailed muscle load model to enable tailored surgical planning based on each patient’s anatomy, tumor location, resection length, and allograft dimensions. This approach embodies personalized medicine, allowing orthopedic surgeons to use CT-based 3D models to predict fixation stability and optimize surgical strategies. It also supports customized postoperative rehabilitation plans aligned with reconstruction stability. Over time, data from such simulations can inform standardized protocols, improving outcomes for bone tumor resection and reconstruction.

## 5. Conclusion

The present study examined plate fixation methods of diaphyseal allografts after intercalary resection of the femur. We found that the four analyzed variants have comparable stress levels in the DFP. The highest stress levels are typically found in the proximal transition area between the allograft and the original bone. Additionally, configurations with supplementary SP showed greater sliding distances at the proximal junction compared to those without SP. In contrast, variants containing PMMA demonstrate substantially decreased sliding distances in the distal junction. Based on the analyzed IFM distances, we recommend the use of a combined plate fixation method with PMMA augmentation as the optimal approach for femoral shaft allograft reconstruction following tumor resection. This technique enhances rigidity, particularly in the distal region, contributing to more stable and durable fixation.

## Supporting information

S1 Fig Screw location map. (JPG)

S2 Fig Results of equivalent stress on the screw number 1. (JPG)

S3 Fig Results of equivalent stress on the screw number 2. (JPG)

S4 Fig Results of equivalent stress on the screw number 3. (JPG)

S5 Fig Results of equivalent stress on the screw number 4. (JPG)

S6 Fig Results of equivalent stress on the screw number 5. (JPG)

S7 Fig Results of equivalent stress on the screw number 6. (JPG)

S8 Fig Results of equivalent stress on the screw number 7. (JPG)

S9 Fig Results of equivalent stress on the screw number 8. (JPG)

S10 Fig Results of equivalent stress on the screw number 9. (JPG)

S11 Fig Results of equivalent stress on the screw number 10. (JPG)

S12 Fig Results of equivalent stress on the screw number 11. (JPG)

S13 Fig Results of equivalent stress on the screw number 12. (JPG)

S14 Fig Results of equivalent stress on the screw number 13. (JPG)

S15 Fig Results of equivalent stress on the screw number 14. (JPG)

S16 Fig Results of equivalent stress on the screw number 15. (JPG)

S17 Fig Results of equivalent stress on the screw number 16. (JPG)

S18 Fig Results of equivalent stress on the screw number 17. (JPG)
