# Supplementary figures and images for: Computational modeling of bone allograft reconstruction following femoral shaft tumor resection: Investigating the impact of supplementary plate fixation
Source: PLoS One. 2025 Feb 6;20(2):e0316719. doi: 10.1371/journal.pone.0316719 (PMC11801617; doi:10.1371/journal.pone.0316719)

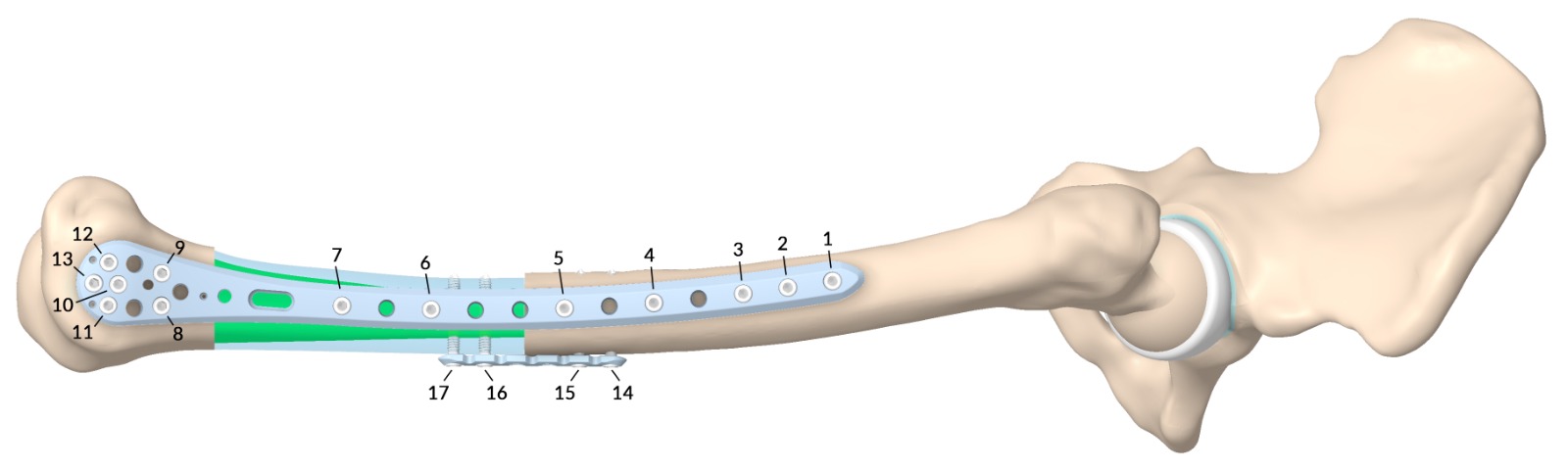

Supplement: S1 Fig — (JPG) [file pone.0316719.s001.jpg]

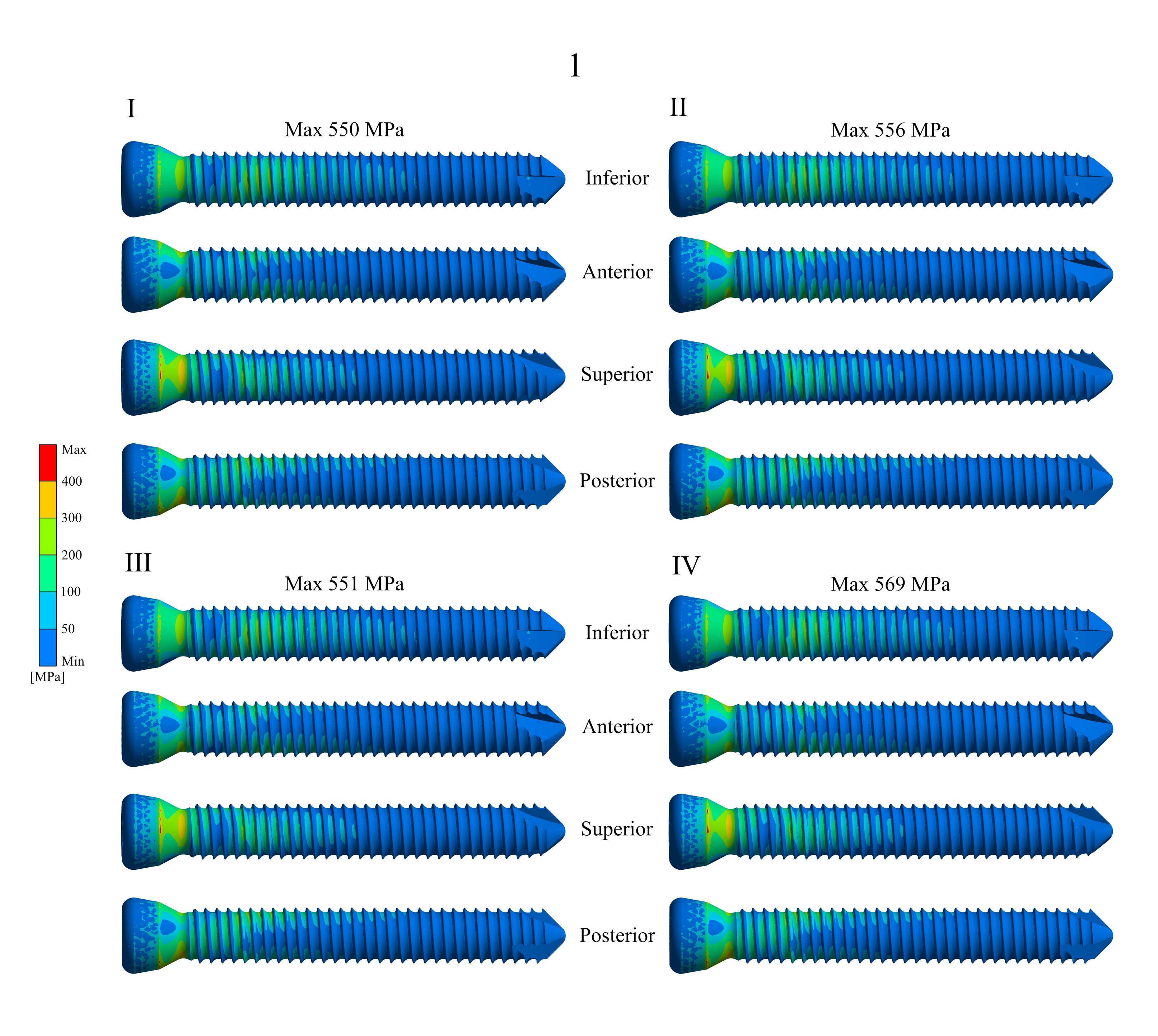

Supplement: S2 Fig — (JPG) [file pone.0316719.s002.jpg]

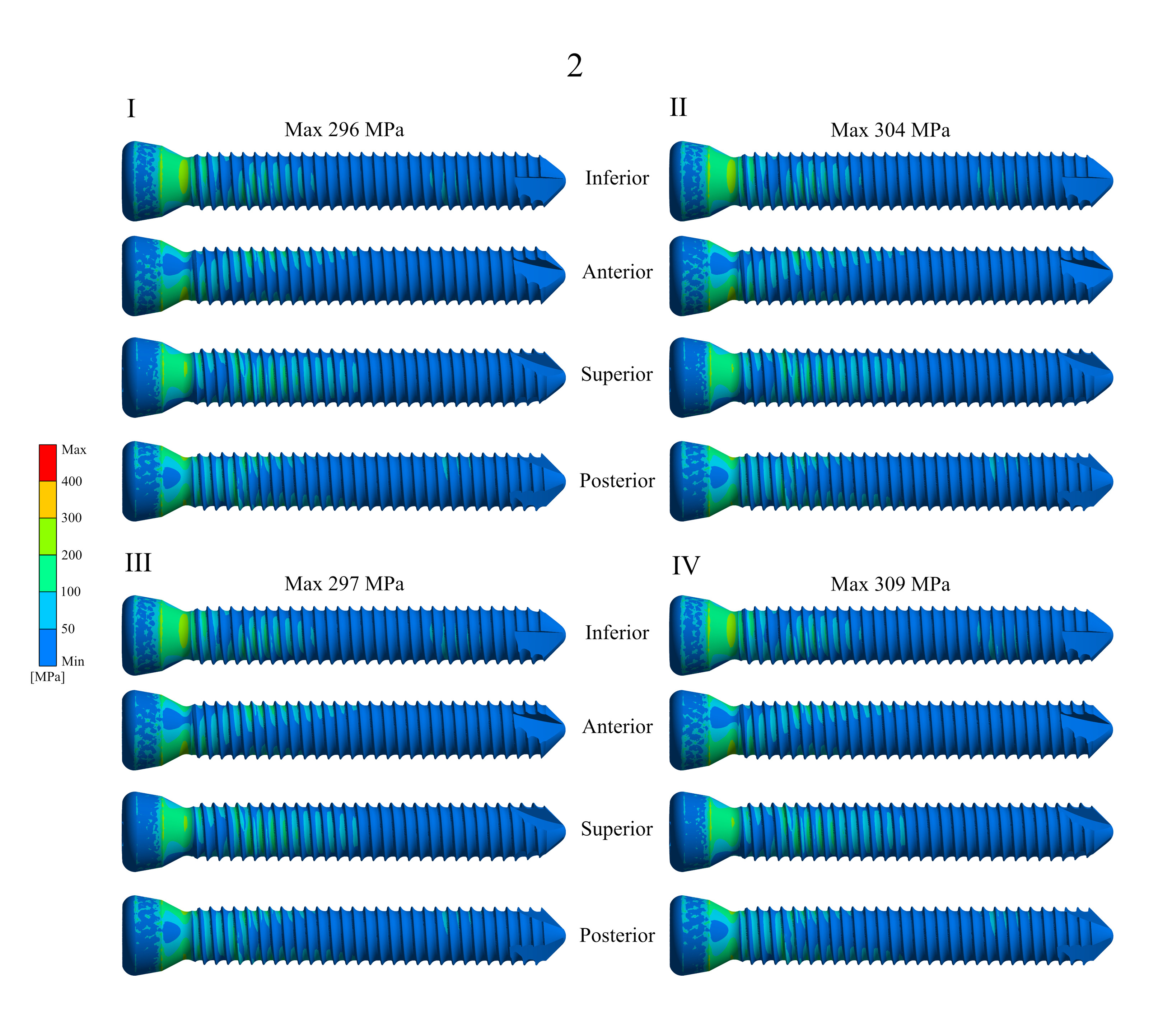

Supplement: S3 Fig — (JPG) [file pone.0316719.s003.jpg]

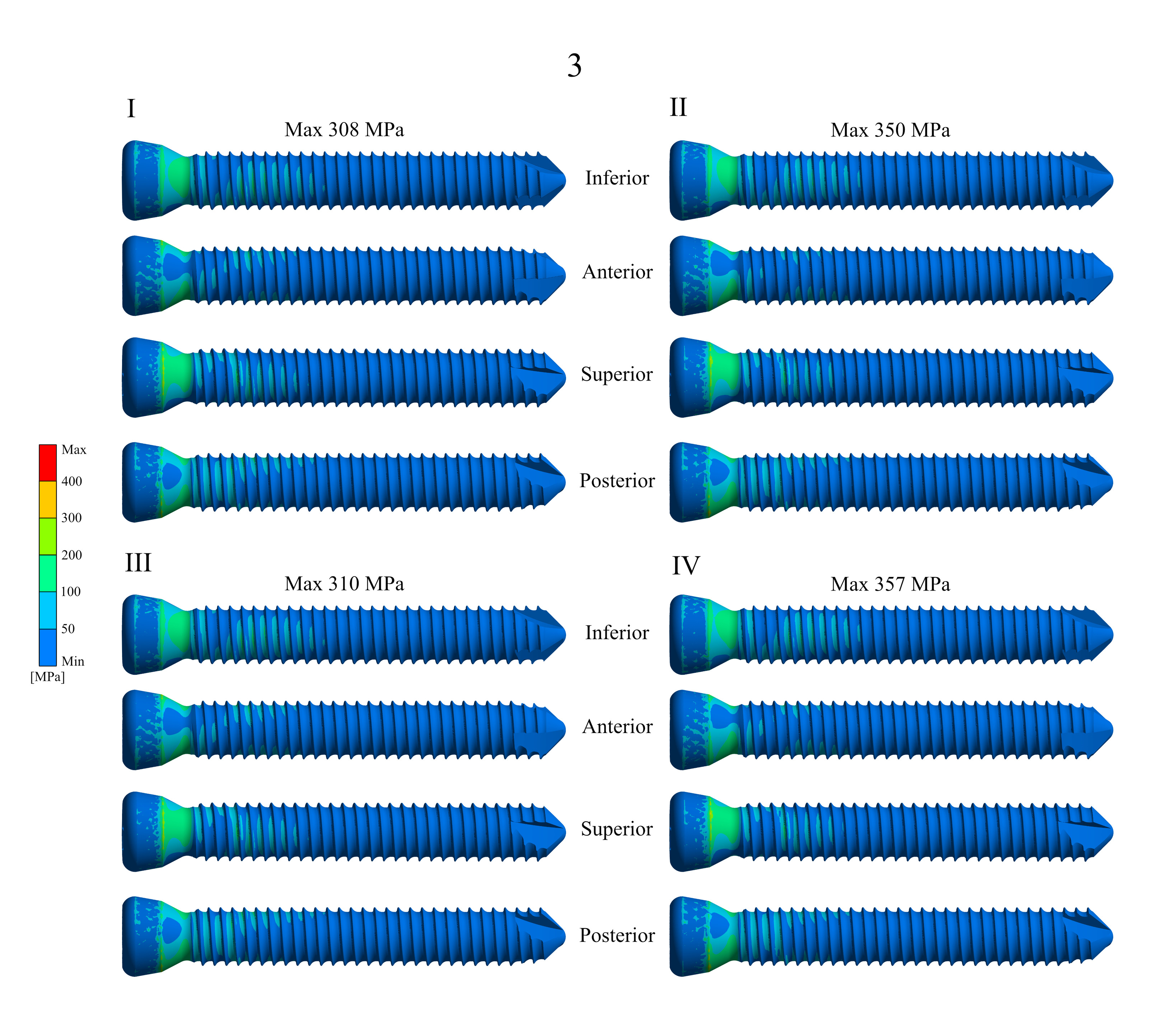

Supplement: S4 Fig — (JPG) [file pone.0316719.s004.jpg]

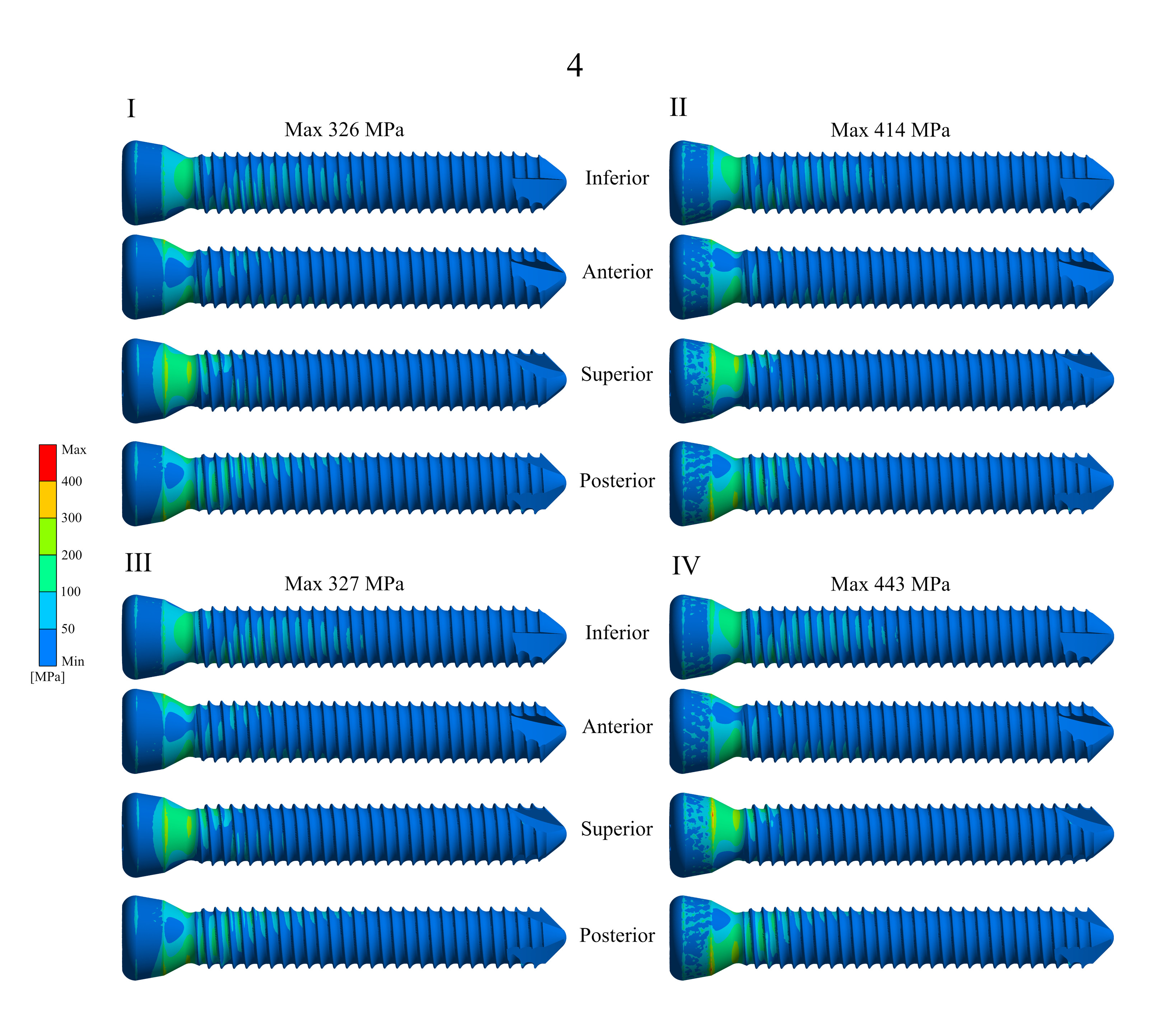

Supplement: S5 Fig — (JPG) [file pone.0316719.s005.jpg]

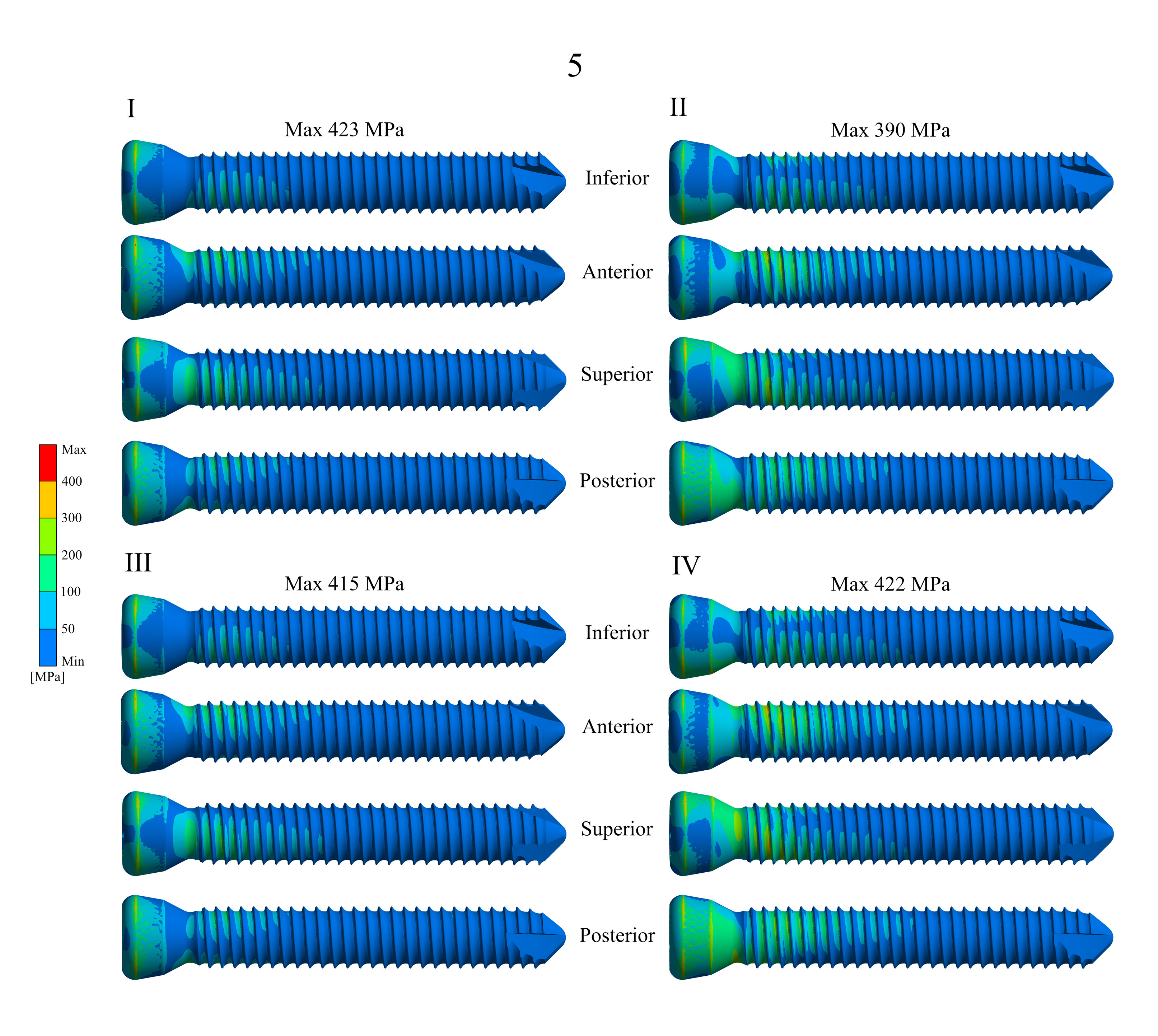

Supplement: S6 Fig — (JPG) [file pone.0316719.s006.jpg]

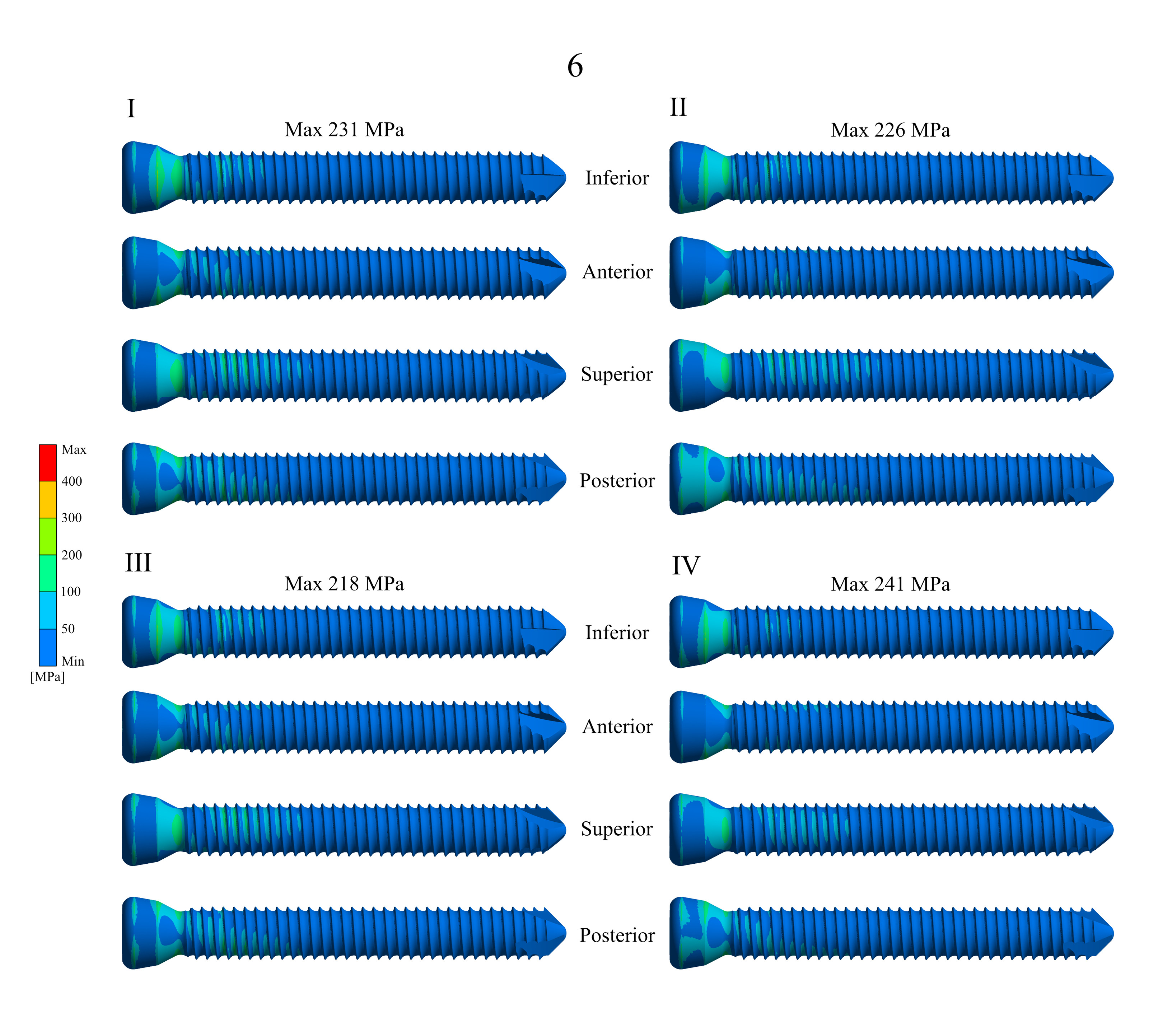

Supplement: S7 Fig — (JPG) [file pone.0316719.s007.jpg]

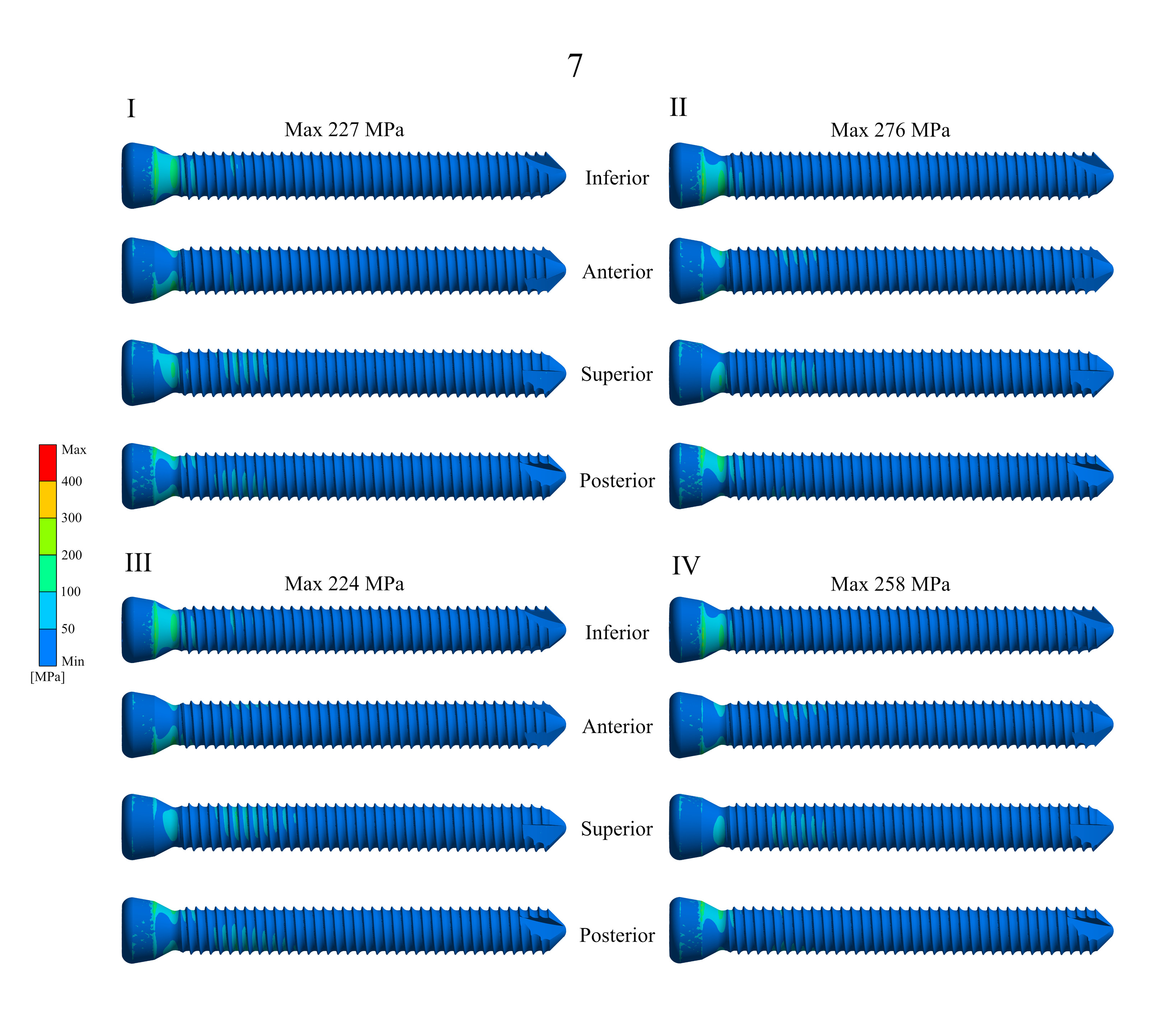

Supplement: S8 Fig — (JPG) [file pone.0316719.s008.jpg]

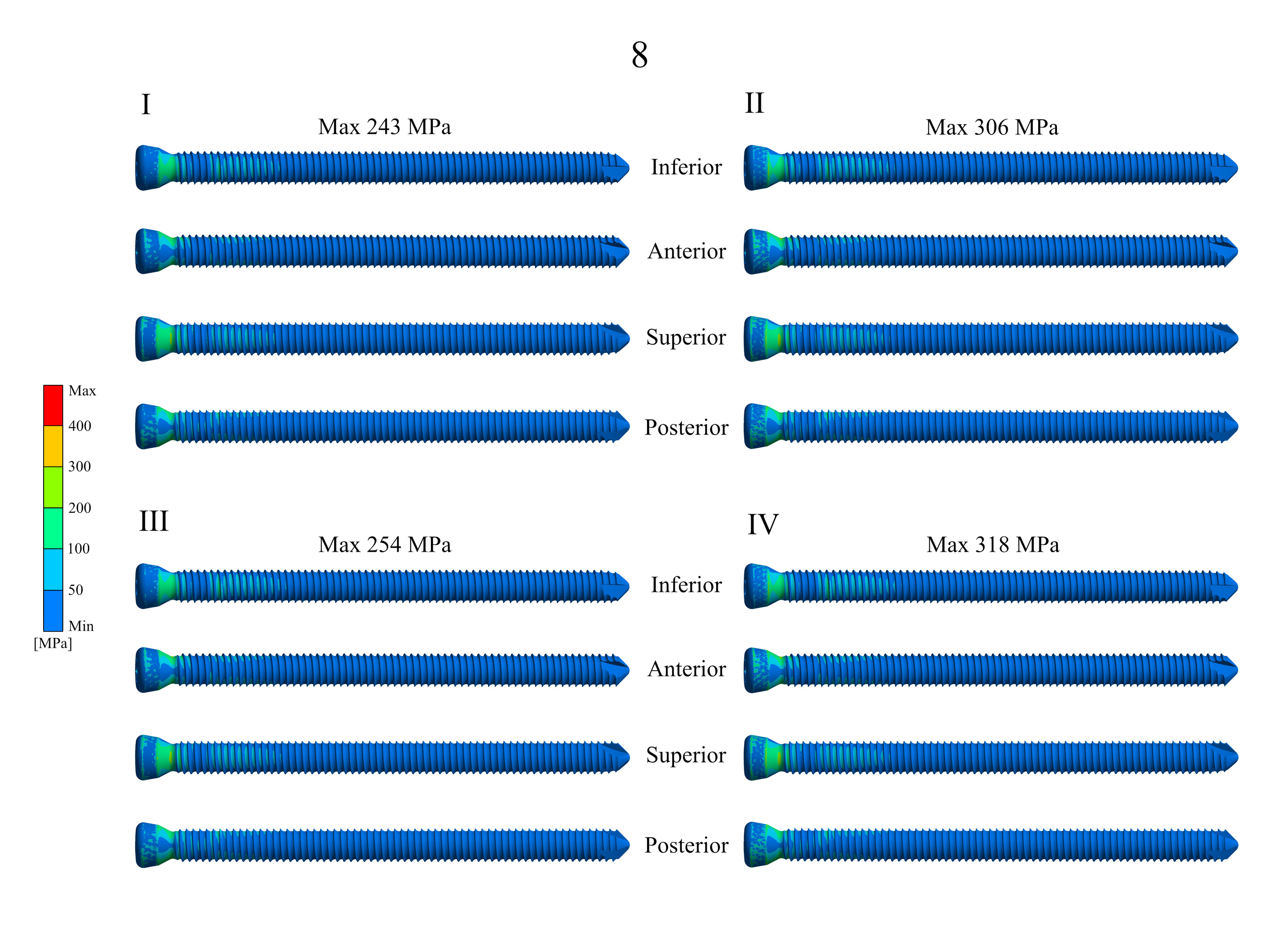

Supplement: S9 Fig — (JPG) [file pone.0316719.s009.jpg]

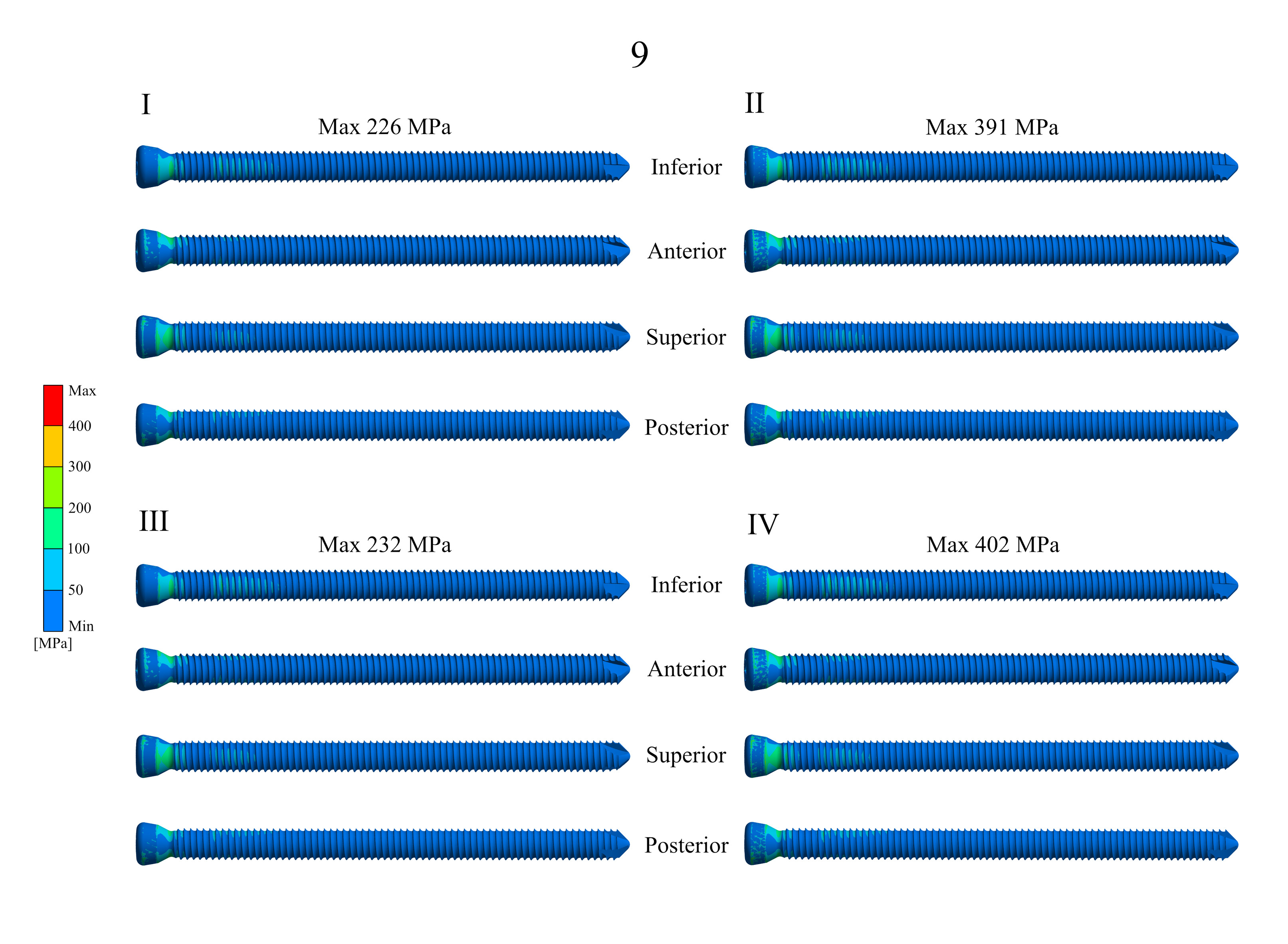

Supplement: S10 Fig — (JPG) [file pone.0316719.s010.jpg]

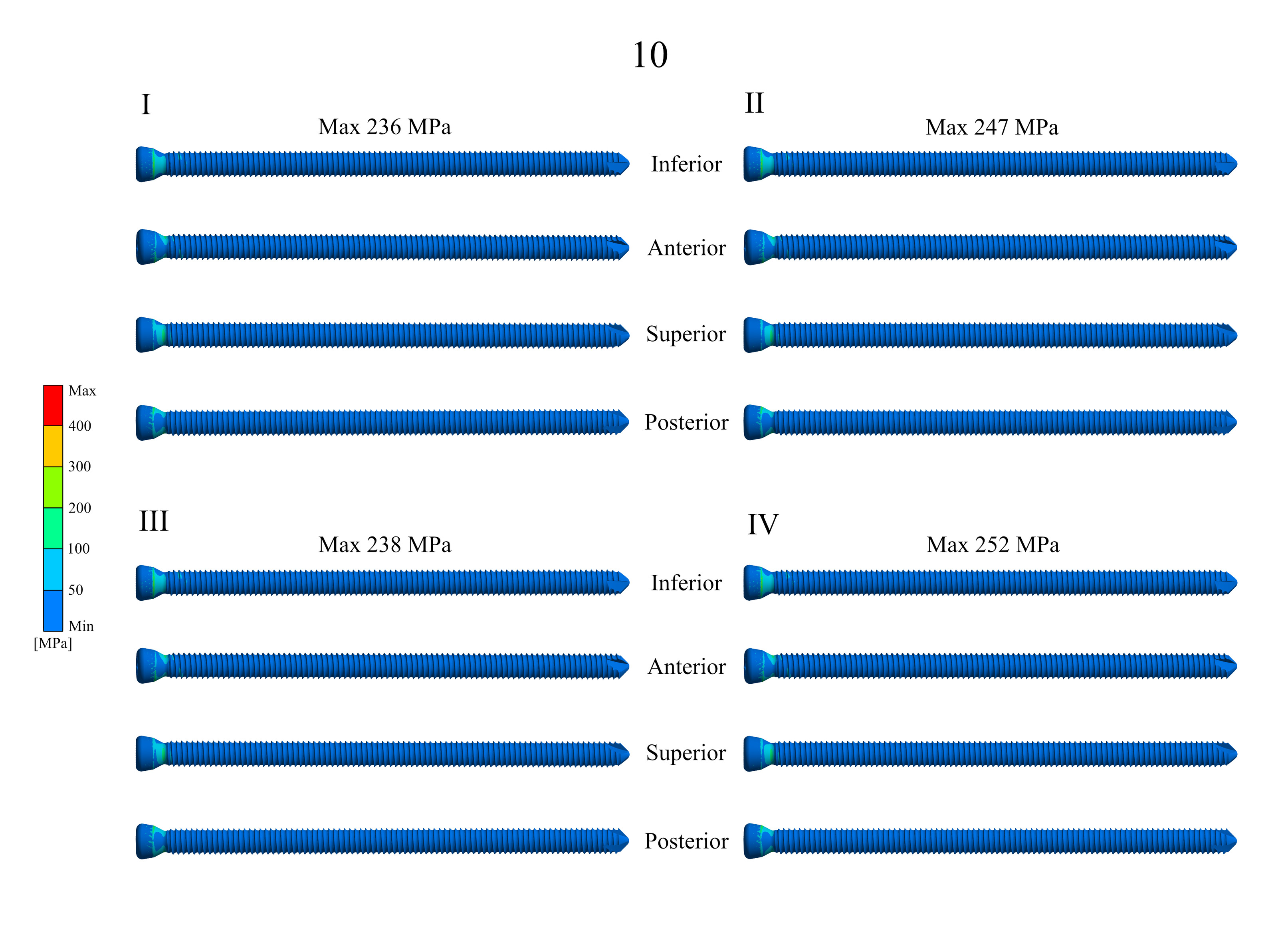

Supplement: S11 Fig — (JPG) [file pone.0316719.s011.jpg]

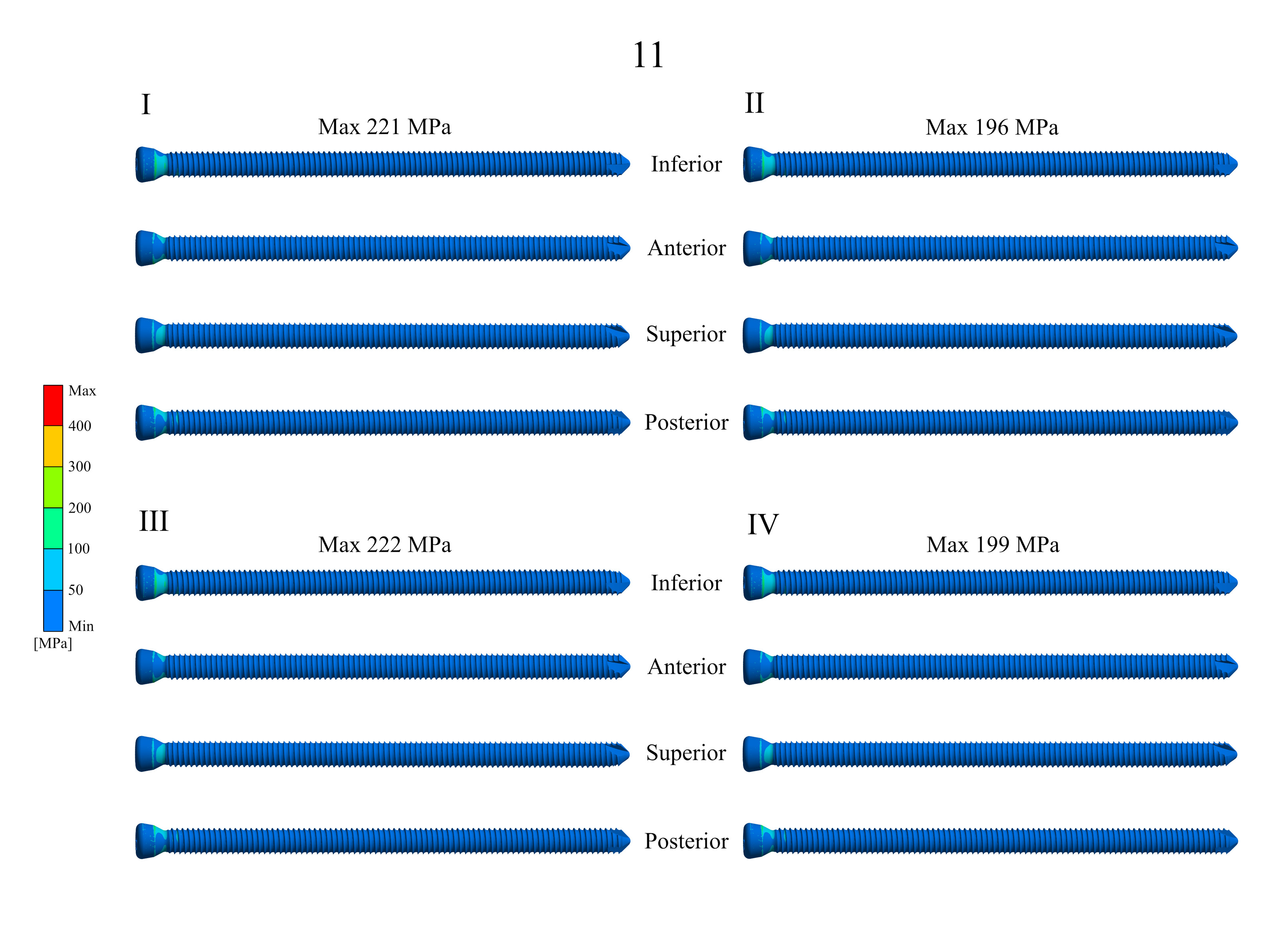

Supplement: S12 Fig — (JPG) [file pone.0316719.s012.jpg]

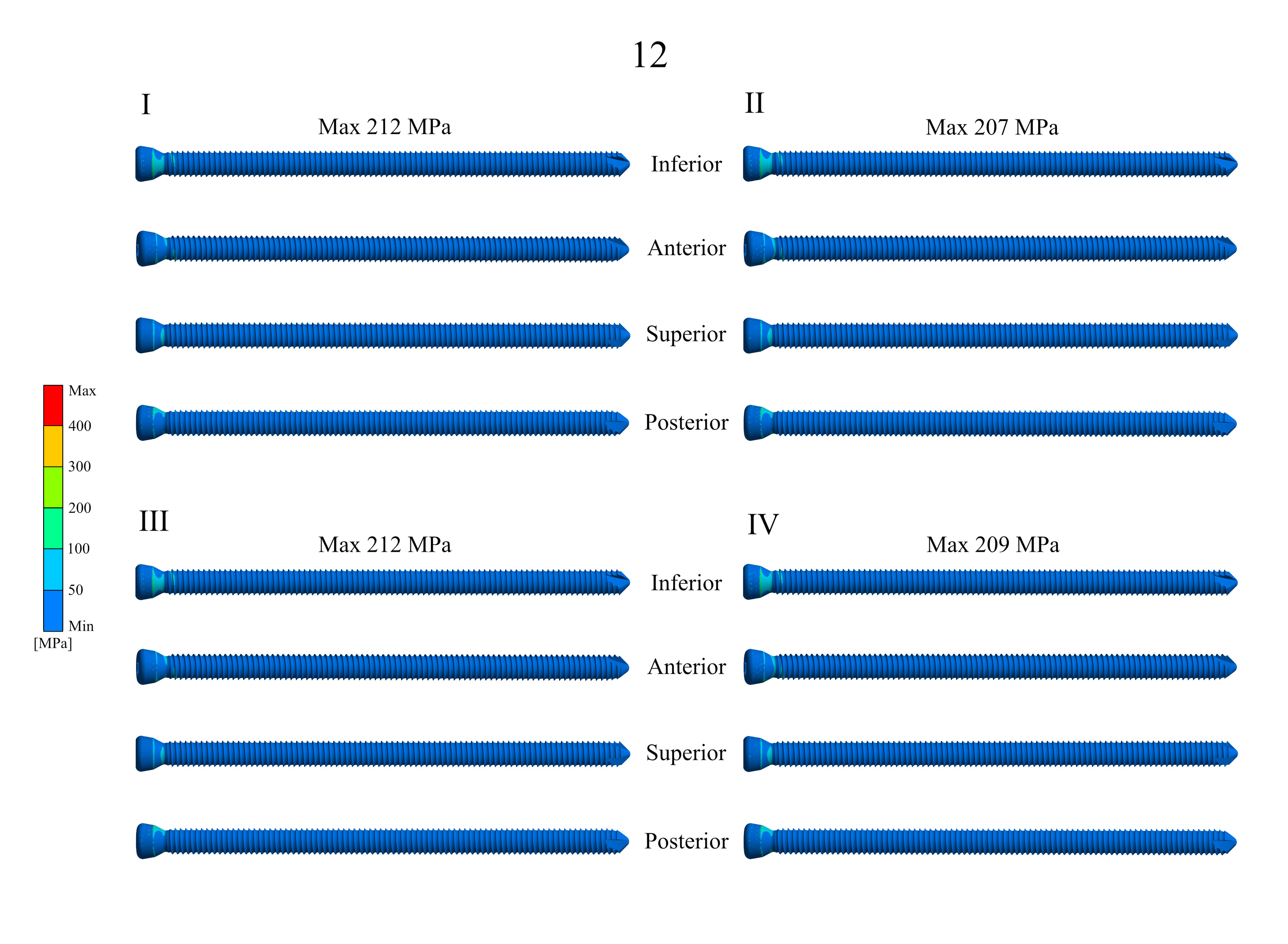

Supplement: S13 Fig — (JPG) [file pone.0316719.s013.jpg]

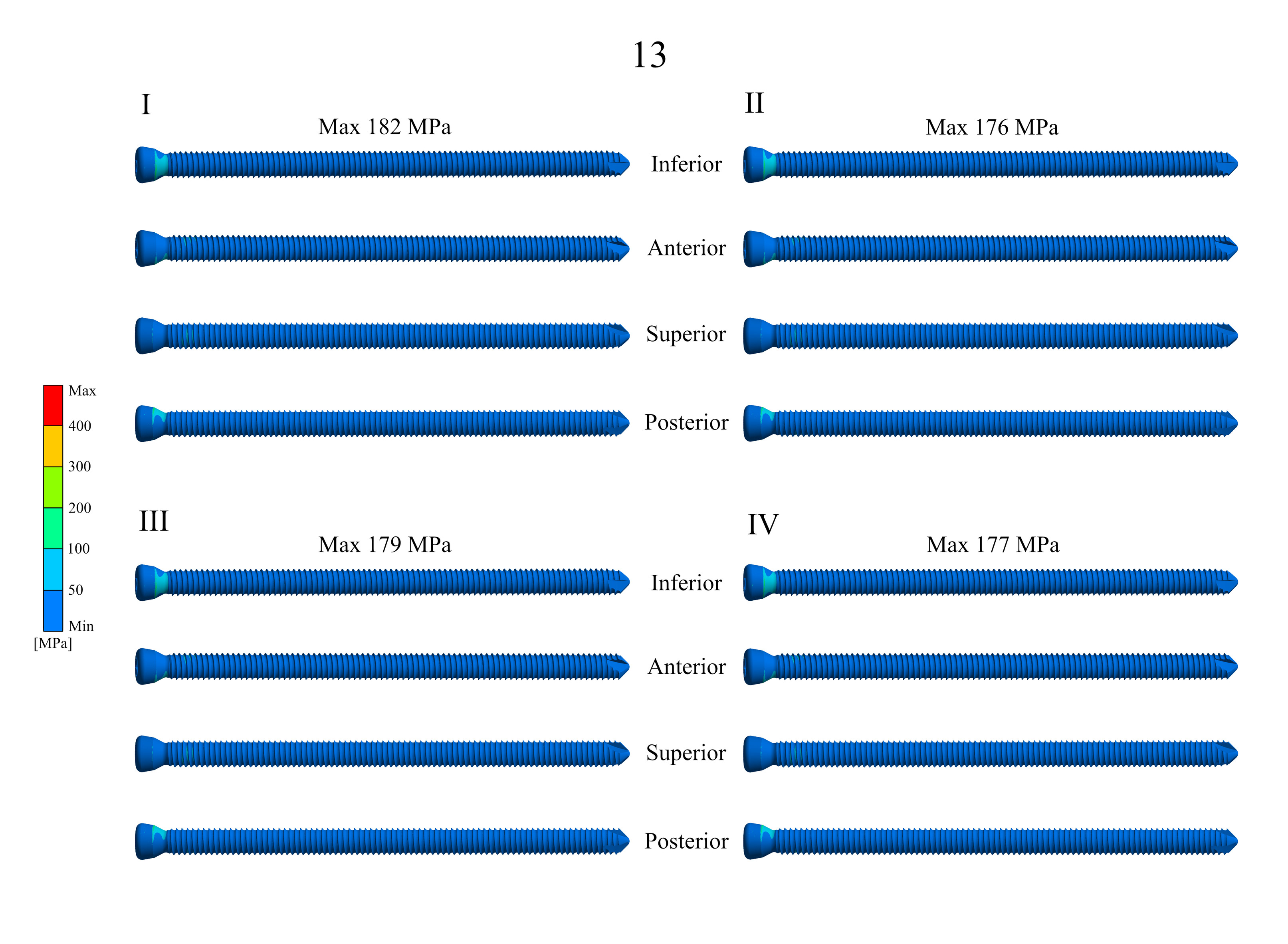

Supplement: S14 Fig — (JPG) [file pone.0316719.s014.jpg]

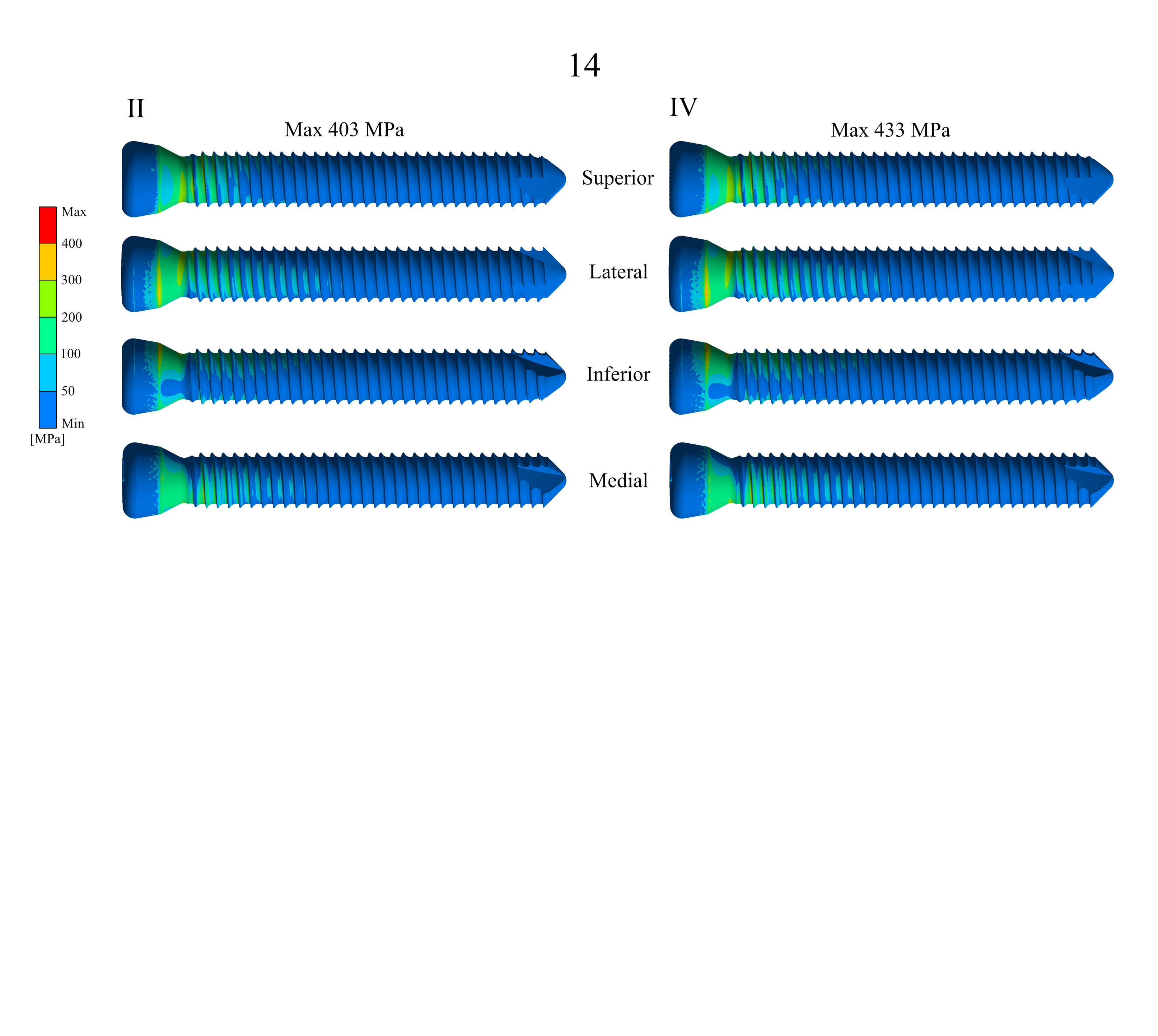

Supplement: S15 Fig — (JPG) [file pone.0316719.s015.jpg]

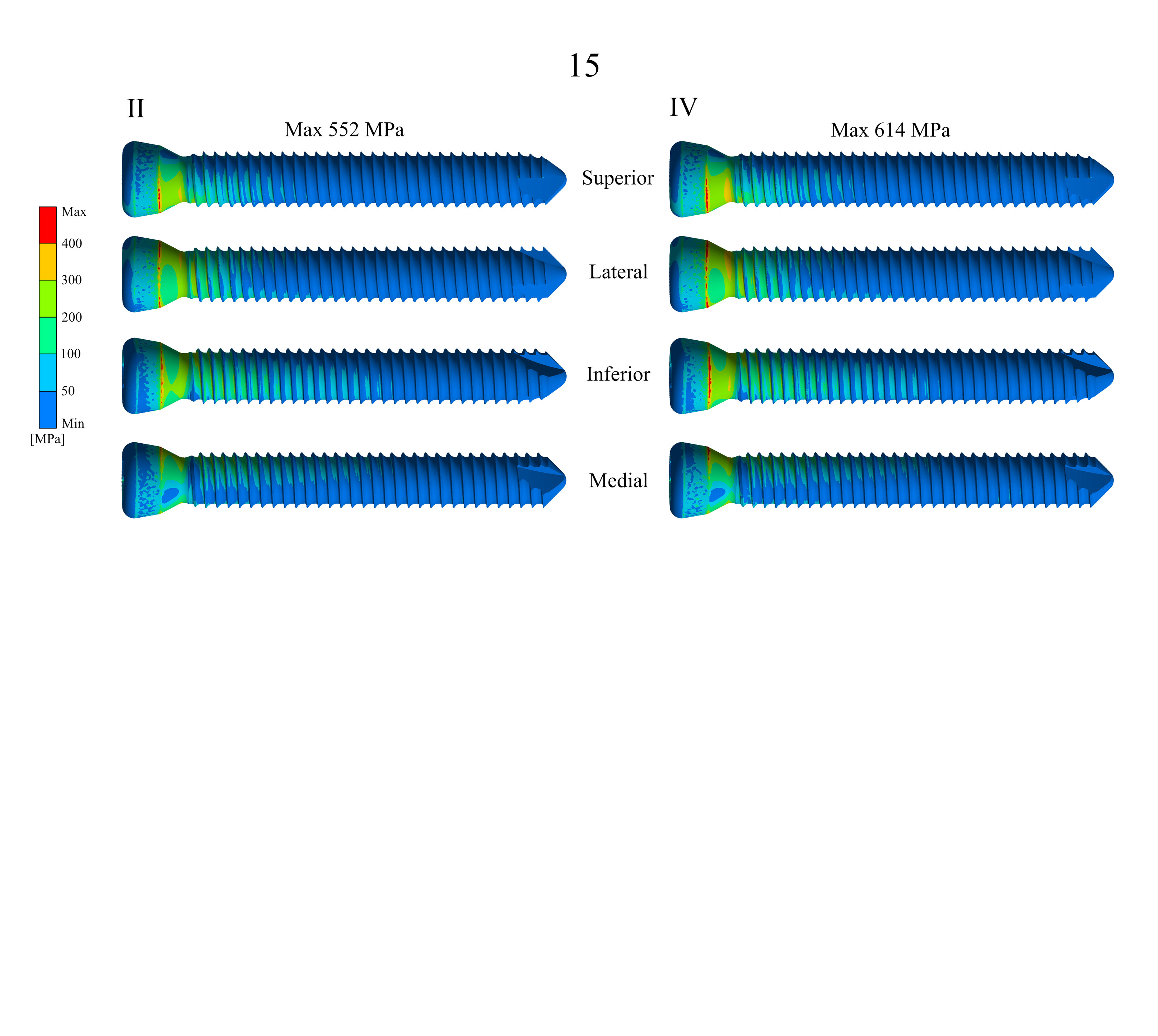

Supplement: S16 Fig — (JPG) [file pone.0316719.s016.jpg]

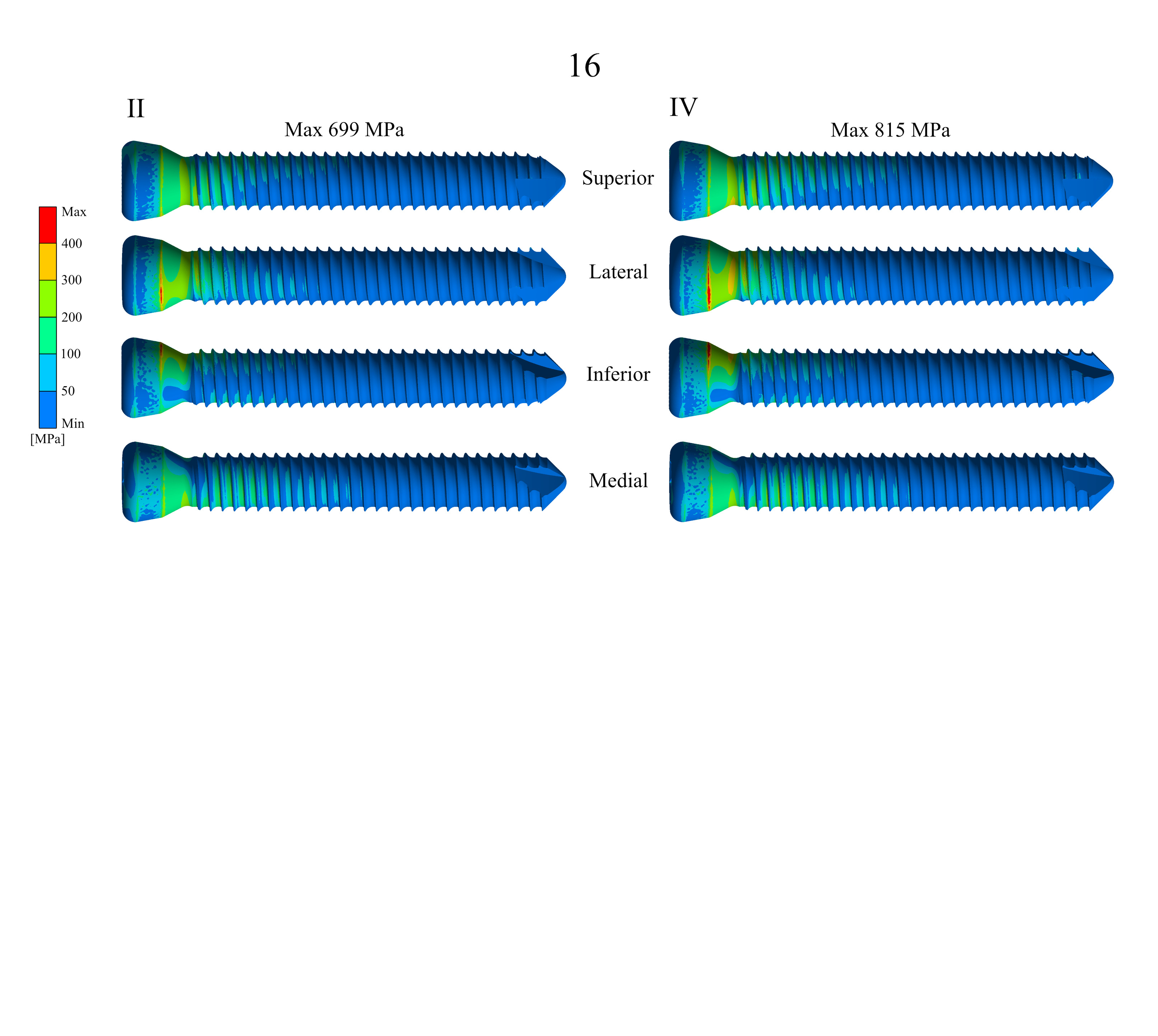

Supplement: S17 Fig — (JPG) [file pone.0316719.s017.jpg]

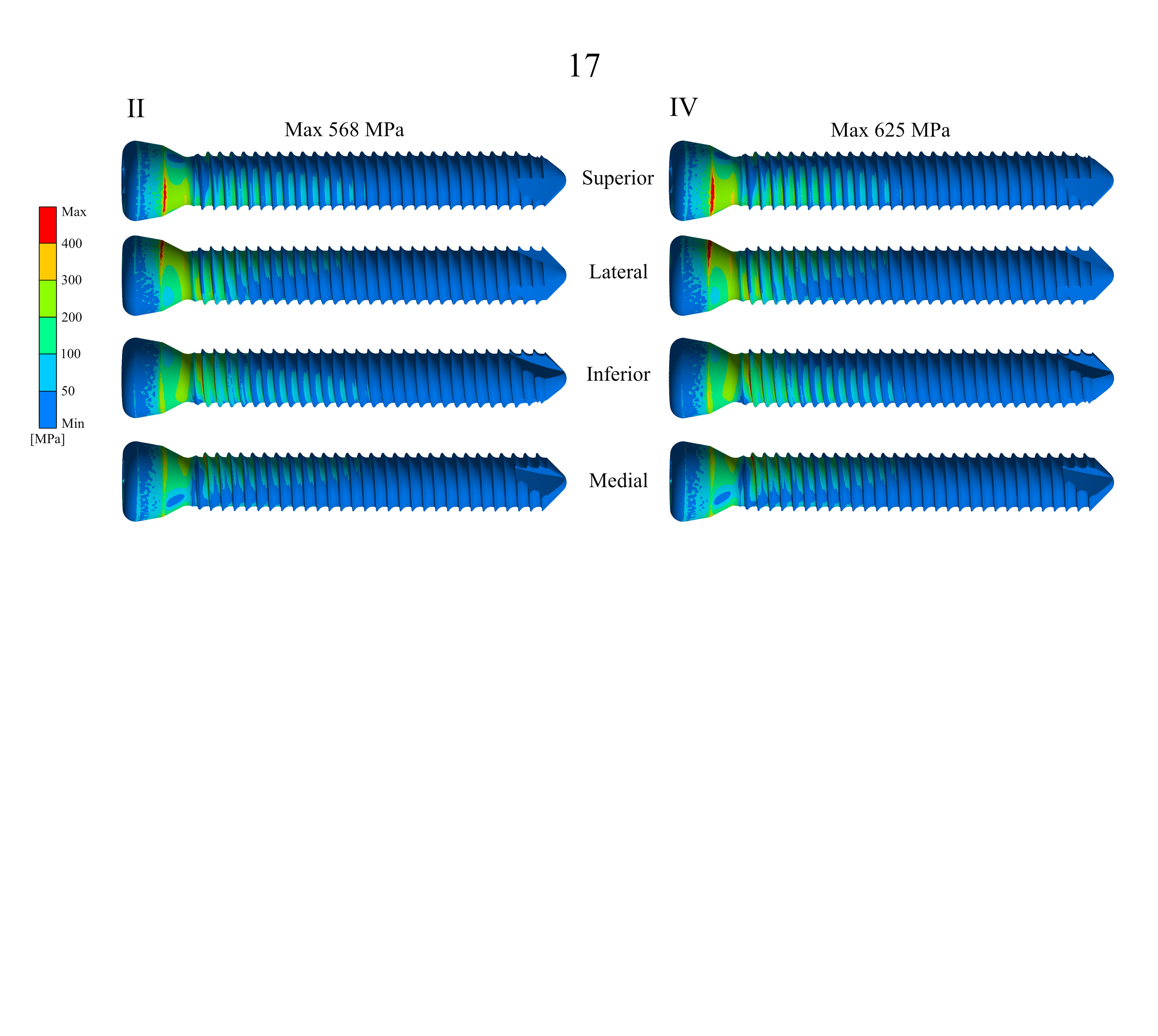

Supplement: S18 Fig — (JPG) [file pone.0316719.s018.jpg]
